# Food contaminants: mechanisms of toxicity, computational assessment, and mitigation

**DOI:** 10.3389/ftox.2025.1719447

**Published:** 2026-01-12

**Authors:** Laura Escorihuela, Rajesh Kumar Pathak, Benjamí Martorell, Vikas Kumar

**Affiliations:** 1 Department of Chemical Engineering, University Rovira i Virgili, Tarragona, Spain; 2 Pere Virgili Institute for Health Research (IISPV), Tarragona, Spain; 3 Department of Pesticides Safety, German Federal Institute for Risk Assessment (BfR), Berlin, Germany

**Keywords:** computational modeling, endocrine disruptors, food contaminants, health risk assessment, toxicity

## Abstract

Understanding the toxicological mechanisms of food contaminants is critical for assessing risks to human health. This review comprehensively examines their adverse effects, tracing the pathway from molecular initiation to systemic organ-level damage. A central focus is placed on the growing trust on computational methods as ethical and practical alternatives to traditional animal testing. The discussion encompasses a multi-scale assessment, detailing atomic-level interactions through Density Functional Tight Binding Molecular docking and Molecular Dynamics (MD) simulations, analyses of toxicity pathway, and prediction of systemic fate using Physiologically Based Pharmacokinetic (PBPK) modeling. We further explore how these *in silico* insights are integrated with experimental data to build predictive models, including Quantitative Structure-Activity Relationship and machine learning frameworks. Ultimately, this review aims to inform the development of effective strategies for mitigating contaminant risks, thereby advancing public health objectives and supporting the 3Rs principles (Replacement, Reduction, and Refinement) in toxicological science.

## 1 Introduction

Chemical contaminants in the food chain, ranging from environmental pollutants to processing by-products, present a persistent threat to human health (Food safety, 2025). Traditional toxicity assessment relying on animal models is increasingly supplemented by advanced *in silico* approaches due to ethical and efficiency demands (Jadhav et al., 2025). This review synthesizes current knowledge on the mechanisms of toxicity of major food contaminants and explores the transformative role of computational tools in predicting their hazard and informing mitigation strategies, thereby shaping a more proactive food safety paradigm (Piparo et al., 2011; Kuppusamy et al., 2024).

Currently, significant efforts are underway in software development to simulate and refine protein interactions in “*in vivo*” media, coupling Molecular Dynamics (MD) and machine learning to predict their stability, interactions and reactivity. As early as 2007, the United States National Research Council proposed that in 21st century, the “*in vivo*” experiments would be substituted by alternative “*in vitro*” assays to assess health risks (Krewski et al., 2010). The effective use of these alternative technologies, such as “*in vitro*” cell tests, hinges on a deep understanding of chemical toxicological mechanisms. This includes comprehending molecular and cellular toxicity pathways, tissue and organ-level toxicity effects, and gathering comprehensive scientific evidence. Ultimately, this understanding facilitates the generation of adverse outcome pathways (AOPs) at both individual and population levels (Escher et al., n.d.). Following this trajectory of toxicological testing development, food toxicology is actively transitioning from traditional animal experiments to novel *“in vitro”* and *“in silico”* strategies (Yang et al., 2023).

Food contaminants originate from diverse sources, including agricultural chemicals, heavy metals from harvesting lands, mycotoxins, and environmental pollutants like dioxins and Polychlorinated Biphenyls (PCBs). All these can enter the body through contaminated food consumption (Tola, 2025). Reflecting a global shift towards ethical testing, the European Union (EU) implemented a comprehensive ban on cosmetics animal testing in 2013, a trend swiftly followed by countries like Norway, New Zealand, and Israel in 2013, and India and Brazil in 2014 (Sreedhar et al., 2020).

The application of computational methods to predict toxicology has surged, driven by the ethical and costs limitations of traditional experiments coupled with ever-increasing computational capacity. These advancements offer quicker insights into society’s growing concerns about food toxicity. Traditional approaches are often constrained by the limited number of doses, time points, organ systems, and combinations that can be realistically tested in a single laboratory experiment. While toxicity regulations demand reproducible assays, experimental methods frequently encounter limitations due to fragmented standardization and inherent variability in conditions, which can be influenced by factors as simple as reagent batch differences or purchase dates.

Advances in computer science promises not only faster experiments but also more robust reproducible results. Furthermore, computational modelling and simulations can connect fields like statistics, chemistry and physics enabling the prediction of outcomes in processing methods with sufficient accuracy to design more effective experimental assays, thereby saving both time and cost (Smith et al., 2022).

Molecular dynamic (MD) simulation stands out as one of the most widely used and promising computational methods (Cavaliere et al., 2020; Zhang et al., 2020). MD simulation has exhibited its outstanding advantages in the design and study of novel drugs, proteins and advanced materials. For instance, MD studies have elucidated the H-bonding interaction between paracetamol and water, revealing how this interaction promotes the solubility of paracetamol (Xu et al., 2018). Sadeghi and co-workers explored the radial distribution functions (RDFs) and spatial distribution functions (SDFs) of ionic liquids *via* MD study (Nian et al., 2021), and suggested that strong interactions between anions and cation can promote anions distributed around cations evenly. Similarly, MD studies exploring the secondary structure and activity of lipase in natural deep eutectic solvents found that lipase can be activated through the H-bond binging interactions (Nian et al., 2021).

In the field of food sciences, molecular simulation technology has mainly been applied to proteins, lipids and carbohydrates. These food components are often combined with other substances and processed to improve their functional properties. MD simulations were first applied to carbohydrates in 1986 by introducing this technology to understand the relationship between carbohydrate structure and its function (Wang et al., 2022). Due to limitations of computer hardware, resources, performance and MD simulation software, the initial simulation could only use 6, 7 or 8 glucose units to represent single chain starch molecules. Related studies showed that the bond length of a virtual angle formed by O4–O4′- O1′ could describe the change in twist angle of glucose residues. Koehler *et al.* in 1988 studied the configuration change of α-cyclodextrin in aqueous solution by MD simulation, but the simulation time was only tens of picoseconds (Wang et al., 2022). Another important factor coupled with the MD simulations is the level of theory used for the calculations of atomic forces, beyond classic force fields. The development and improvement of electronic structure calculation methods based on or derived from quantum mechanics, as is the case of the Density Functional Tight Binding (DFTB) theory, and the ever-growing computational capabilities in supercomputing in recent times, makes it possible nowadays to simulate dynamics models that were unthinkable a few years ago (in DFTB one can easily scale up to 5,000–10,000 atoms systems using) (Çetin et al., 2024). Such techniques, which are 3,000 to 30,000 times faster than regular Density Functional Theory (DFT), allows considering more realistic sizes and conditions such as molecules solvation or molecule adsorption on surfaces. In recent years, some of the aforementioned techniques based on MD have been successfully applied to investigate nanosized titania with sizes 2.2–4.4 nm (Mikolajczyk et al., 2023).

For the evaluation of food toxicity, we must focus our attention on particles added to food as ENMs. Physicochemical characteristics of ENMs are the key determinants of their techno-biological functionality, as well as the source of potential adverse health effects. A list of such physicochemical properties has been defined by the Organisation for Economic Co-operation and Development (OECD). Amongst these parameters the size, chemical composition aggregation agglomeration state, and surface treatment/coating of ENMs appear to be most critical for nanotoxicity issues. Parameters that can be perfectly estimated by computational methods within the MD framework.

In this review, we aim to present the different tools based on MD to study fundamental atomic interaction and simulate the interaction of ENMs and other common particle present in food with proteins and other biomolecules. We will examine MD techniques such as DFTB, Molecular docking and Virtual Screening, where protein–small-molecule interactions are essential for the sustainability of biological processes such as enzymatic catalysis and overall homeostasis in the body. These is a good technique to analyze the architecture of compounds generated by two or more distinct molecules. Additionally, we will analyze the interaction between chemical compounds and their target protein receptors at the atomic level, which is an effective tool in drug design and provide a good estimation of the binding (Maden et al., 2022; Keval and Tejas, 2022). Also, we want to mention Molecular dynamics simulations and MM-PBSA, a popular approach to estimate the free energy of the binding of small ligands to biological macromolecules (Genheden and Ryde, 2015; Tuccinardi, 2021), by combining molecular mechanics calculations and continuum solvation models (Hou et al., 2011). It is more computationally efficient than the related approach, the linear interaction energy (LIE) method, which averages interaction energy from the MD simulations to estimate the absolute binding free energy. It has been successfully applied to various protein−ligand (Hou et al., 2002; Stoica et al., 2008) or protein−protein/peptide complexes (Hou et al., 2006).

We want to go a step further in the field of toxicology and the calculation of physical and chemical properties with MD. These can subsequently be used as descriptors for Quantitative Structure-Activity Relationship (QSAR) toxicology models or to feed new models based on Artificial Intelligence. The proposed computational approach is in line with the 3R principles (Reduction, Replacement and Refinement) for animal testing and contributes to the development of alternate testing methods and the implementation of intelligent testing strategies (Escorihuela et al., 2018a). The OECD has established principles and guidance for validating and using QSAR models for regulatory purposes, ensuring they are scientifically robust and reliable. This guidance has led to the increasing acceptance of QSAR predictions in various regulations related to chemical substances, including those under REACH (Registration, Evaluation, Authorisation and Restriction of Chemicals) ((Q)SAR Assessment Framework: Guidance for the regulatory assessment of (Quantitative) Structure Activity Relationship models, predictions, and results based on multiple predictions Series on Testing and Assessment No. 386, 2023).

To provide a comprehensive understanding of how chemical substance moves through the body (absorption, distribution, metabolism, and excretion) by incorporating physiological and biochemical information we must also mention the Physiologically based pharmacokinetic (PBPK) models (Pletz et al., 2020), PBPK models can incorporate various levels of physiological complexity and drug elimination and distribution processes depending on the intended use and the available knowledge of the drug’s characteristics (Isoherranen, 2025).

## 2 Engineered nanoparticles and other contaminants in food

According to the European Food Safety authority. NM refers to any intentionally produced material that has one or more dimensions of the order of 100 nm or less in one or more dimensions, composed of discrete functional parts and including structures, agglomerates or aggregates, which may have a size above the order of 100 nm but retain properties characteristic of the nanoscale. Moreover, ENM that contain <50% nanoparticles by number are not considered nano by certain regulatory agencies (e.g., European Food Safety Authority, EFSA), who is the responsible for the risk assessment of the use of nanomaterials in food and feed, as well as in food contact materials, other agencies such as Food and Drug administration in EUA (FDA), do not have a percentage in the specifications (Sohal et al., 2018).

The physicochemical properties of nanomaterials can differ significantly from those of their bulk counterparts. Although these differences confer important technological advantages, ENMs may also pose potential risks to human health and the environment. Evaluating their potential hazards requires a thorough characterization of surface structure, physicochemical properties, and reactivity, as these factors determine how ENMs interact among them and with environmental components. In particular, surface characteristics, tendency of nanoparticles to aggregate, which directly affects their environmental fate and exposure potential. Moreover, aggregation can alter surface properties, thereby modifying interparticle interactions and influencing overall reactivity (Mikolajczyk et al., 2023).

The physicochemical properties in general, as size, roughness, surface area of NPs have a great impact on their toxicity since they can change the mechanism of toxicological response given accumulation, uptake, and trans-location inside the cell, by organelle or membrane uptake. One single material with different shapes and sizes can considerably change the response of live tissue and identify NPs as a safe or toxic (Attarilar et al., 2020).

For instance, size-related changes in particles smaller than 5 nm are more pronounced than those observed in particles ranging from 15 to 90 nm, primarily due to quantum size effects. One particularly relevant phenomenon associated with smaller NPs is their ability to penetrate biological systems directly, where they can dissolve and release toxic metal ions—a mechanism known as the “Trojan horse” effect (Stenzel, 2021). This effect is specific to nanoscale materials and results from their inadvertent recognition and uptake by cellular receptors (Escorihuela et al., 2018a).

The European Union Observatory for nanomaterials (EUON) (https://euon.echa.europa.eu/food1) also has a specific webpage for food and food packaging, novel food and even for Nanomaterials in active and intelligent food contact materials where we can find EU regulations. *EUON* published on 22 October 2024, reviewed the release of nanoparticles from consumer products, including from food contact materials (FCMs), and their potential toxicity (Abram et al., 2025).

Food industry uses ENM for multiple functional applications to enhance flavours, colours, as food additives, food packaging, as antimicrobials for improving food preservation, for nutrient encapsulation and enhancing bioavailability, and in agricultural practices to enhance food quality, safety, nutrient delivery, and production yield (Zhang L. et al., 2025).

Accordingly, the current *EUON* report finds that applications of nanomaterials in packaging have significantly increased in the past decades increasing the human exposure and related impacts. For FCMs, the concern pertains to nanoparticles migration into the food, leading to human exposure. While the nano-size and high surface area-to-volume ratio of the particles bring advantages to materials such as resistance, flexibility, or thermal conductivity their small size also has drawbacks, such as the ability to cross biological membranes (Gupta et al., 2024).

For instance, SiO_2_ is used as colour additives, flavours, packaging, and anticaking agents in powdered foods. Nanoparticles are also effective carriers for fragrances and flavoring agents. In addition to flavour enhancement, ENMs are also employed to improve food appearance, for instance, titanium dioxide (TiO_2_) is approved as a food colorant, with regulatory limits typically set below 1% w/w. TiO_2_ is also used as a food additive and flavour enhancer in a variety of non-white foods, including dried vegetables, nuts, seeds, soups, and mustard, as well as beer and wine. Titanium dioxide in nanoform is an antimicrobial agent, sometimes in combination with other compounds or elements as nickel oxide and cobalt, for the inactivation of foodborne pathogens. TiO_2_ also serves as an active component in packaging due to its inert nature and low toxicity. Mechanistic studies indicate that under ultraviolet (UV) irradiation, TiO_2_ generates reactive oxygen species (ROS), including hydrogen peroxide, hydroxyl radicals, and superoxide anions. These species can disrupt microbial cell walls, providing a potent antimicrobial effect (Sharma et al., 2023; Ameta et al., 2020).

The properties of ENMs enable their applications as food additives, functional food carriers, in active and intelligent packaging materials, and in agricultural practices to enhance food quality, safety, nutrient delivery, and production yield. In solution, nanoparticle-based sensors, array biosensors, electronic noses, nano-test strips, and nanocantilevers are among the different types of nanosensors used for packaging (Biswas et al., 2022; Anjum et al., 2023). These include sensors capable of detecting foodborne pathogens such as *Escherichia coli* and *Salmonella*, or the controlled release of antimicrobial agents (Suvarna et al., 2022). For example, silver-based nanocomposites have a strong antibacterial activity. However, zinc oxide (ZnO) nanoparticles are considered significantly less toxic to humans and animals than silver nanoparticles (Ag NPs), and thus offer a safer alternative. ZnO is frequently used in cosmetics, medical devices, drug delivery systems, UV light absorbers, active packaging, additive in supplements, antimicrobial, and antifungal. The United States Food and Drug Administration (FDA) currently recognizes ZnO as “generally recognized as safe” (GRAS) (Espitia et al., 2013).

Another example of nanoparticles used in food industry are Carbon NanoTubes (CNTs), used as a nanosensors, active packaging, antibacterial or antifungal, they absorb undesirable flavors, as well as in low-resistance conductors and catalytic reaction vessels, gelation, and as viscosifying agent (Ashfaq et al., 2022; Fuciños et al., 2019).

Iron, in the form of iron oxide, is mostly used as a food colorant (Sieg et al., 2024). Iron nanoparticles are a health-promoting food additive since iron deficiency is one of the most common micronutrient deficiencies worldwide. The solubility and the bioavailability of poorly acid-soluble iron compounds can be improved by decreasing their primary particle size and thereby increasing their specific surface area (Hilty et al., 2011; Hilty et al., 2011; Hilty et al., 2011; Hilty et al., 2011).

For instance, SiO_2_ nanoparticles, or silica nanoparticles have been reported to act as effective carriers for fragrance and flavoring agents, anti-caking agent or stabilizer (Zhang et al., 2023).

Another important effect of ENM is the nano-encapsulation, which involves the incorporation, absorption or dispersion of bioactive compounds in or on nano-sized vesicles. This may protect the bioactive compounds against degradation, improve stability and solubility (e.g., solubilizing a hydrophilic compound in hydrophobic matrices and *vice versa*) leading to increased bioavailability and delivery to cells and tissues (Peters et al., 2016; Peters et al., 2016; Peters et al., 2016; Peters et al., 2016; Mallia et al., 2022).

Due to the specific ENM physicochemical properties and high reactivity, ENM can influence basic cellular processes, such as proliferation, metabolism, and death. A report by the British Royal Society notes that we may face a nanotoxicity crisis in the future (The Royal Society et al., 2004). Only with a proper detailed understanding of the properties of nanomaterials like size, solubility, surface chemistry, composition, *etc.*, we will be in a position to find useful and safe food products. Exist a gap in the lack of Reference Materials (RM) or reference test materials (RTMs) for NM characterization in complex media such as food, consumer products and other matrices. Current RMs fail to reflect the complexity of these materials, making it difficult to ensure consistent and accurate testing across different types of NMs and NM matrices, thus hindering innovation and compliance (Abram et al., 2025).

To make us and idea about the use on ENM in food we can check the data available in the Nanotechnology Product Database (https://product.statnano.com/) where are reported the use of nanomaterials in food products by countries, among many other data available to evaluate.

On the other hand, we focused also with BPA, that contaminates food through migration from polycarbonate plastic containers and the epoxy resin linings of metal food cans. This endocrine disruptor is linked to health problems including diabetes, obesity, and potential issues with reproduction and the brain. The values of BPA in food in Europe has been changed from 2025 by the EFSA, this agency has lowered the tolerable daily intake (TDI) for BPA respect to 2015, around 20,000 times lower (Bisphenol A in food is a health risk|EFSA, 2023).

## 3 Molecular mechanisms of toxicity

The physicochemical properties of nanomaterials and reactivity can differ significantly from those of their bulk counterparts. Although these differences confer important technological advantages of ENMs may also pose potential risks to human health and the environment.

Evaluating their potential hazards requires a thorough characterization of surface structure, physicochemical properties, and reactivity, as these factors determine how ENMs interact with one another and with environmental components (Mikolajczyk et al., 2023).

Recent studies have provided deeper insights into the size-dependent behaviour of nanoparticles, demonstrating that smaller nanoparticles (NPs) tend to show greater variability in their properties and reactivity, whereas larger NPs exhibit more consistent behaviour (Faria et al., 2015). One particularly relevant phenomenon associated with smaller NPs is their ability to penetrate biological systems directly, where they can dissolve and release toxic metal ions—a mechanism known as the “Trojan horse” effect. This effect is specific to nanoscale materials and results from their inadvertent recognition and uptake by cellular receptors (Escorihuela et al., 2018b).

For non-nano contaminants like mycotoxins, pesticides, and industrial chemicals such as BPA and its analogs, toxicity primarily arises from compound-specific mechanisms. These include metabolic activation (Heikkinen et al., 2025), receptor-mediated disruption of endocrine signaling (Tang et al., 2025), and direct macromolecular damage. The Organisation for Economic Co-operation and Development (OECD) defined a list of physicochemical properties to assess the nanotoxicity of nanomaterials that can be determined by MD simulations. Amongst those parameters one finds the size, chemical composition, aggregation agglomeration state, and surface treatment/coating of ENM appear to be most critical for nanotoxicity issues. Due to the specific ENM physicochemical properties and high reactivity, ENM can influence basic cellular processes, such as proliferation, metabolism, and death. Individual ENMs may lead to one or more toxicity endpoints, resulting in dysfunction of these basic processes. In recent years, several *“in vitro”* studies have assessed the potential adverse health effects of ENMs, and the main toxicity mechanism identified are:

### 3.1 Ability to induce oxidative stress species (ROS)

The most relevant pathogenetic pathway linking ENM exposure to tissue damage is represented by the induction of reactive oxygen species (ROS) generation (Liu et al., 2013). Oxidative stress arises when there is an imbalance between (ROS) production and the body’s ability to neutralize or repair the damage caused by these species. The ENM contaminants often induce ROS production, which can harm cellular macromolecules such as lipids, proteins, contributing to diseases such as inflammation in tissues or even cell death. Additionally, ENM can affect immune responses by altering immune cell function, leading to immune suppression or autoimmune disorders (Gosslau, 2016; Tola, 2025). ROS are highly reactive molecules—this includes moieties such as the superoxide anion (O^2−^), hydrogen peroxide (H_2_O_2_), and hydroxyl radicals (OH^·^)—and play a crucial role in cellular signalling, but have the potential of being harmful when generated excessively. They release toxic ions and cause the oxidative stress, disrupt electron/ion cell membrane transport activity and cause oxidative damage and lipid peroxidation, whereas results from *“in vivo”* studies have shown that these materials can induce adverse effects on the respiratory, cardiovascular and nervous systems. Literature demonstrated that ZnO NP and Ag NP used as antimicrobial to food packaging and carbon nanotubes (CNT) can elevate intracellular ROS levels triggering oxidative stress ractions (Zhang L. et al., 2025). ROS also induce to cellular disfunction damaging lipids, proteins, and even DNA.

Other common toxicity endpoints involve cytotoxicity and genotoxicity (Martirosyan and Schneider, 2014). Prolonged oxidative stress has been linked to chronic inflammation, which plays a crucial role in the pathogenesis of various diseases, including cancer, neurodegeneration, and cardiovascular disorders (Batir-Marin et al., 2025). Studies have shown that zebrafish embryos exposed to Ag NPs, TiO_2_ NPs, and CdSe quantum dots exhibit significant generation of ROS, leading to apoptosis and developmental abnormalities (Batir-Marin et al., 2025). Reduced NP size correlates with increased ROS generation and toxicity in aquatic organisms, including zebrafish. For instance, silver nanoparticles (Ag NPs) smaller than 10 nm trigger higher oxidative stress levels compared to larger particles due to their enhanced cellular penetration and bioavailability (Flores-López et al., 2019). Similarly, titanium dioxide (TiO_2_) NPs of <20 nm induce significant ROS production and mitochondrial dysfunction in zebrafish embryos (Faria et al., 2015). Another example is SiO_2_, where the toxicity of SiO_2_ nanomaterial has been evaluated “*in vitro*” and “*in vivo*” (Perez-Borm et al., 2006; OECD, 2004) no acute toxicity of amorphous SiO_2_ after conducting a toxicity study. But a recent study showed that fumed SiO_2_, a form of synthetic amorphous silica, produced reactive oxygen species (ROS) and caused red blood cell hemolysis (Yang et al., 2016).

And other recent work conclude that cytotoxicity and organ damage caused by SiO_2_ NPs vary and are affected by many factors, including the SiNP type, particle size, pore size, degree of modification, dosing frequency, dosage, and administration time. And the toxicity mechanisms of this NPs include oxidative stress, inflammation, and apoptosis; however, research on the involved signal pathways remains relatively incomplete (Huang et al., 2022).

### 3.2 Surface charge and coating, surface reactivity

The surface charge of ENMs affects their interaction with biological membranes and proteins. The size of nanoparticles is a very important feature, crucial role in their toxicity potential, for its unique properties as the surface area of engineered nanoparticles (ENPs) depends upon its size (Ameta et al., 2020). Small NPs exhibit higher surface-area-to-volume ratios, increasing their reactivity (Batir-Marin et al., 2025). In general, positively charged ENPs interact more easily with cell membranes due to electrostatic attraction to the negatively charged cell membranes, leading to increased cellular uptake and higher cytotoxicity compared to neutral or negatively charged ENMs (Zhang L. et al., 2025). Surface coatings are applied to ENPs to modify their properties, such as improving stability, dispersibility, and biocompatibility. Common surface coatings include polymers (e.g., Poly-ethylene-glycol or PEG), surfactants (e.g., Tween 80), and inorganic materials (e.g., silica). These coatings can alter interactions with biological systems, potentially influencing their toxicological profiles. For instance, PEG modification, is often employed to reduce toxicity. One study demonstrated that PEG-coated Ag NPs exhibited lower cytotoxicity, immune activation, and tissue damage compared to uncoated Ag NP (Bastos et al., 2016). When NPs enter biological environments, such as zebrafish media or blood plasma, they rapidly adsorb proteins and other biomolecules on their surfaces, forming a dynamic protein corona. This corona can significantly alter nanoparticle properties, including surface charge, hydrodynamic diameter, colloidal stability, and cellular interactions, ultimately affecting biodistribution, oxidative stress responses, and toxicity profiles (Liu et al., 2025).

### 3.3 Solubility

Solubility is another key physicochemical property that warrants particular consideration and impacts ENM endpoint toxicity, as it affects how they interact with cells, tissues, and the environment. Insoluble or partially soluble ENMs in the digestive fluids, draw more attention due to their potential to cross the gastrointestinal tract as an intact particle. As stated by the EU Scientific Committee on Emerging and Newly Identified Health Risks (SCENIHR), free and low solubility ENMs are a priority concern for human and environmental safety (Martirosyan and Schneider, 2014).

When nanoparticles finally dissolve, they can release toxic ions or change their size, surface area, and overall chemical behavior (Eisenbrand, 2020a). Solubility and speed (rate) of dissolution are dependent on a particle’s chemical and surface properties, as well as size, and are further impacted by the surrounding media. The potential for NPs to dissolve can effectively influence their persistence in the environment and act as a critical control on their biological response. Although size is considered as the primary physicochemical property affecting solubility of NPs, various other parameters such as, surface area, surface morphology, crystallinity and crystal structure also need to be considered, as the strength of the surface bonds, their spatial arrangements as well as the presence of impurities, and storage conditions may influence NPs dissolution (Misra et al., 2012; Eisenbrand, 2020b). For example, if NPs aggregate and sediment, they become available to sediment dwelling organism and can enter through dietary uptake.

Similarly, if the NPs dissolve in the exposure media, speciation of ions with other ligands can be prominent and the uptake mechanism of ions in organisms will be different. NPs that resist complete dissolution in the media can have a combination of possible routes, *viz.* Endocytosis of NPs, or ion transportation of the dissolved components or a combination of both. There may be another scenario, where NPs associated with the biological membrane can act as a reservoir of metal ions, which are released at a variable rate when NPs undergo dissolution. It is also worth noting that studying NPs’ dissolution is not only important for correct interpretation of nano-toxicological data, but can also help in the current move towards “safety by design of NPs” For example, surface functionalization of carbon nanotubes (e.g., using PEG) can facilitate their solubility and thus reduce their biopersistence and any potential toxicity (Misra et al., 2012). It is generally assumed that solubility may increase as particle size decreases, as described by Ostwald–Freundlich equation (Equation 1), correlating interfacial tension and solubility:

$$\frac{s}{s\left(bulk\right)}=\exp\left(\frac{4\gamma V}{RTd}\right)$$ (1)

Equation 1. Ostwald Freundlich equation. Where s is the solubility (mol·kg^−1^) of spherical particles, d (m) diameter, g (mJ·m^−2^) is the interaction energy, V is the molar volume (m^3·^mol^−1^), R gas constant (mJ·mol ^−1^ K ^−1^) and T(K) temperature (Escorihuela Martí, 2019).

In the case of CuO NPs, experimental data shows an increase in dissolution (rate of dissolution and equilibrium concentration) with reduction in particle size. For ZnO NPs, on the other hand, does not seem to be any significant difference in dissolution between nanoparticles and bulk particles (micron sized particles). Ag NPs present a more complex scenario, which is primarily because size control in the case of metallic Ag NPs is often achieved using surface modifications (Misra et al., 2012). Depending on the NPs’ status within the surrounding media with respect to their dissolution behaviour (i.e., which of the following combinations will be present: NPs/ions; ions/complexes; suspended/agglomerated NPs) their bioavailability, uptake rates and toxicity will vary, if NPs aggregate and sediment on release, they become available to sediment dwelling organism and can enter through dietary uptake.

### 3.4 Genotoxicity and DNA damage

Genotoxicity refers to the capability of chemical agents to damage DNA, leading to mutations, chromosomal aberrations as well as disrupted cellular processes (Kopp et al., 2018; Woo et al., 2011). Food contaminants exert genotoxicity through reactive metabolites, oxidative stress and direct binding to the DNA that will leads to the serious health effects (Payros et al., 2017; Woo et al., 2011). Contaminants present in food such as mycotoxins including aflatoxin B1 and ochratoxin A (Corcuera et al., 2015), by products of processed food such as acrylamide (Eisenbrand, 2020b), heavy metals like arsenic, cadmium, and lead, pesticide residues, and environmental pollutants are responsible for genotoxicity (Dimitrijević et al., 2023; Salvagni et al., 2011; Sherif et al., 2023). MD simulations revealed dynamic aspects of guanine-TiO2 interactions, suggesting surface-induced chemical transformations such as guanine dehydrogenation catalytically mediated by the surface, which may contribute to genotoxic effects (Çetin et al., 2024). Additionally, emerging threats such as nanoplastics and BPA alternatives have become a growing concern in recent years (Sendra et al., 2023; Tang, 2025).

### 3.5 Endocrine and receptor-mediated effects

Another ENM public health concern is their ability to disrupt endocrine functions through receptor-mediated pathways (Peivasteh-roudsari et al., 2023), (Chemical contaminants in food and feed|EFSA, 2021) These endocrine-disrupting chemicals (EDCs) interfere with hormone signalling even at low exposure levels, contributing to diverse health issues ranging from developmental disorders to cancer (Ahn and Jeung, 2023; Özel and Rüegg, 2023). Food contaminants or toxic chemicals present in food can exert their effects through interactions with nuclear receptors (Paramasivam et al., 2024). These interactions can alter the normal function of the receptors, leading to various diseases. Key nuclear receptors include, among others, estrogen receptors, androgen receptors, and pregnancy X receptor (Paramasivam et al., 2024; Ahn and Jeung, 2023; Liang et al., 2023).

BPA is a well-recognized food contaminant and endocrine disruptor found in the environment. It binds to multiple nuclear receptors, disrupting their normal functions (Gokso̷yr et al., 2024; Yuan et al., 2023). In addition, other food contaminants such as polychlorinated biphenyls (PCBs), dioxins, and pesticides can also affect nuclear receptors and their functions. Furthermore, BPA alternatives used in industry may also interact with nuclear receptors (Gokso̷yr et al., 2024; Troisi et al., 2020; Pathak et al., 2024; Pathak and Kim, 2024). These interactions are associated with several health issues, including neurodevelopmental, reproductive, metabolic, and other hormone-related disorders (Ahn and Jeung, 2023). Additionally, food contaminants can cause non-receptor-mediated effects by inhibiting enzymes, altering DNA methylation, and affecting cell proliferation and metabolism through activation of G protein-coupled estrogen receptors (Ahn and Jeung, 2023).

### 3.6 Immune disruption and apoptosis

Food contaminants have a major impact on the immune system and cellular integrity, making them a serious and expanding hazard to human health (Zahir et al., 2025). These environmental pollutants affect apoptosis and impair immunological function, which leads to a variety of issues related to health (Zhang Y. et al., 2024; Suzuki et al., 2020; Winans et al., 2011). Aflatoxins play a significant role in cancer development by affecting various biochemical and molecular pathways. These involve causing direct DNA damage and mutations, and responsible for suppressing apoptosis, weakening the immune system, and triggering epigenetic changes (Mafe and Büsselberg, 2024; Dai et al., 2022).

2,3,7,8-Tetrachlorodibenzo-p-dioxin (TCDD) is one of the most well-studied immunotoxicants, and early research established its key role in shaping the field of developmental immunotoxicology (Winans et al., 2011). Exposure to TCDD during pregnancy has been shown in rodent studies to alter immune development in the offspring (Vos and Moore, 1974; Winans et al., 2011). TCDD-exposed offspring also exhibit weakened responses to influenza virus, including reduced clonal expansion of effector lymphocytes, lower IFNγ levels, and reduced antibody production (Vorderstrasse et al., 2004; Vorderstrasse et al., 2006; Winans et al., 2011). Additionally, polychlorinated biphenyls (PCBs) and polycyclic aromatic hydrocarbons (PAHs) show immunotoxic potential based on human epidemiological data (Winans et al., 2011). Research suggests that inappropriate activation of the aryl hydrocarbon receptor (AhR) pathway by environmental toxicants such as TCDD, PCBs, and PAHs disrupts normal immune system development (Winans et al., 2011).

## 4 *In silico* approaches for food contaminant risk assessment

**FIGURE 1:**
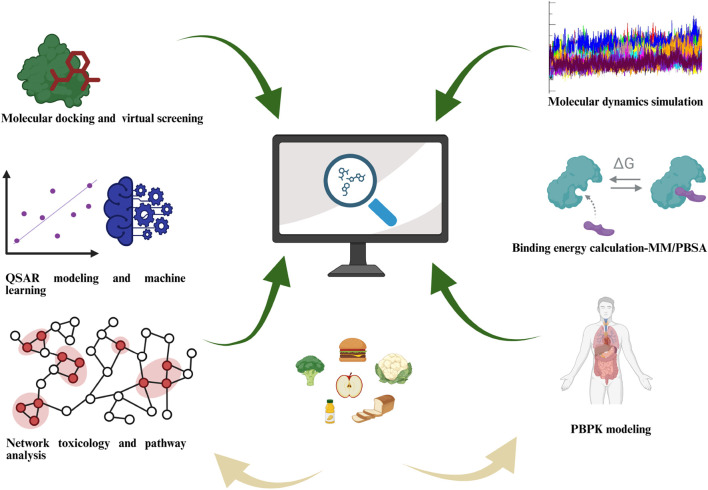
Key computational methodologies for *in silico* toxicology and risk assessment of food contaminants. The depicted approaches form a complementary toolkit: molecular docking, QSAR, and machine learning enable high-throughput hazard prioritization; network toxicology elucidates mechanistic pathways; while molecular dynamics with MM/PBSA provides detailed biophysical validation. These inform PBPK models for quantitative exposure and risk prediction, building an integrated animal-free safety assessment strategy.

In silico approaches provide valuable tools for evaluating the possible risks of food contaminants (Raies and Bajic, 2016). Techniques like molecular docking and virtual screening help researchers predict how contaminants might bind to biological targets, offering early clues about their toxicity (Trisciuzzi et al., 2018). Molecular dynamics simulations take this further by modelling how stable these contaminant-target complexes are and how they behave over time (Salo-Ahen et al., 2020). To measure how tightly they bind, methods like MM/PBSA calculate binding free energy, giving solid way for better risk assessment (Chen et al., 2018). Additionally, physiologically based pharmacokinetic (PBPK) modeling predicts how contaminants are absorbed, distributed, metabolized, and excreted (ADME) in the body (Sharma et al., 2018). This allows for thorough risk assessments without needing animal tests. A schematic of the computational risk assessment paradigm for food contaminants is presented in Figure 1. The graphic integrates the initial exposure *via* foodstuffs with the subsequent multi-scale modeling approach from atomistic simulations to population-level tools. To provide a comprehensive overview of the computational approaches employed in modern risk assessment, we have compiled a list of representative software tools in Table 1. This table lists commonly used applications, categorizing them by their primary function in the workflow, including molecular docking, molecular dynamics simulations, binding energy calculations, and physiologically based pharmacokinetic (PBPK) modeling. Tools for regulatory-oriented tasks, such as the OECD QSAR Toolbox, and for systems-level analysis, like network analysis software are also included. This compilation serves to contextualize the diverse toolkit available to researchers and frames the methodological landscape within which our subsequent analysis is situated. A critical awareness of each method’s predictive value and limitations, validated where possible with experimental evidence, supports their effective application in food safety.

**TABLE 1:** Summarizes key computational platforms used for prediction, evaluation, and risk assessment of food-derived contaminants and toxins.

S.No.	Software name	Role in risk assessment	Example use in food contaminant assessment	References
1.	AutoDock vina	AutoDock Vina helps investigate the binding energy of toxins with human target receptors and determine their interaction mechanisms. It also helps in visualizing toxin–receptor interactions.	Docking of aflatoxin B1 or other contaminants to liver receptors	(Eberhardt et al., 2021) (Iori et al., 2024)
2.	Glide	Glide predicts toxin-receptor binding energy with accuracy, provides mechanistic insights, and enables detailed visualization of docking poses.		(Friesner et al., 2004; Desmond|Schrödinger Life Science, https://www.schrodinger.com/platform/products/desmond/)
3.	Gromacs	Gromacs is used to simulate how toxins bind to receptors, revealing changes in receptor stability, conformational dynamics, and binding interactions over time.	MD simulation of BPA with nuclear receptor to study binding stability.	(Páll et al., 2020; Li et al., 2015; Abraham et al., 2015)
4.	Desmond	Desmond enables high-performance molecular dynamics simulations to analyze the stability, conformational flexibility, and interaction profile of toxin–receptor complexes over time.		(Desmond|Schrödinger Life Science, https://www.schrodinger.com/platform/products/desmond/)
5.	g_mmpbsa	Used to estimate the binding free energy of toxins using MM-PBSA calculations on trajectory data generated from Gromacs simulations.	Calculating the binding energy of BPA and other food contaminants with human receptors	(Cherian et al., 2020; Mengeling et al., 2025)
6.	gmx_mmpbsa	gmx_MMPBSA is a tool used to calculate the binding free energy of toxins using trajectory data generated from Gromacs. It supports both MM/PBSA and MM/GBSA methods.		Valdés-Tresanco et al. (2021)
7.	OECD QSAR Toolbox	It is a computational platform for predicting toxins using read-across, QSAR models, and to fill data gaps.	Profiling of food contaminants for toxicity analysis	(Kutsarova et al., 2021) (OECD QSAR Toolbox|https://www.oecd.org/en/data/tools/oecd-qsar-toolbox.html)
8.	Cytoscape	Cytoscape is used to visualize and analyze molecular interaction networks, helping identify key targets involved in toxin-induced health risks.	Analyse the carcinogenic mechanisms of food contaminants such as herbicide residues through network toxicology	(Cytoscape: An Open Source Platform for Complex Network Analysis and Visualization, n.d.; Otasek et al., 2019; Chen et al., 2025)
9.	MATLAB	Matlab’s SimBiology toolbox enables PBPK modeling to simulate toxin distribution across tissues, supporting pharmacokinetic analysis.	Risk assessment of chemical contaminants such as drug residues in food.	(SimBiology - MATLAB, https://es.mathworks.com/products/simbiology.html) (Dede et al., 2018)
10.	PK-Sim	It is a PBPK modeling software that simulates toxin distribution, metabolism, and elimination across tissues to predict pharmacokinetic profiles.		(Kuepfer et al., 2016; Niederalt et al., 2018; Zhang et al., 2025b; Mi et al., 2025)

### 4.1 Molecular docking and virtual screening

Molecular docking is a powerful computational technique used to predict interactions between a ligand and its receptor (Vidal-Limon et al., 2022; Okus et al., 2024). It estimates the binding energy of a ligand with a receptor and helps to understand the strength and nature of their interaction. In contrast, virtual screening allows the simultaneous evaluation of multiple ligands or ligand database with a receptor, making it valuable for identifying potential lead compounds (Agnihotry et al., 2020; Pathak et al., 2020). While molecular docking has traditionally been applied in drug discovery research, it is now frequently used in the field of toxicology to assess health risks by predicting how toxins may interact with human receptors (Zhao et al., 2025). In recent years, molecular docking and virtual screening has emerged as a transformative computational tool for elucidating interactions between food contaminants such as nanoparticles and chemical toxins with biological targets. This approach accelerates risk assessment by predicting molecular interactions and prioritizing toxic compounds based on their binding free energy for downstream analysis (Kar et al., 2024; Chu and Zi, 2024). Chu and Zi used molecular docking and network toxicology approaches to identify six core protein targets such as Serine/Threonine Kinase 1 (AKT1), Epidermal growth factor receptor (EGFR), Proto-oncogene tyrosine-protein kinase Src (SRC), Tumor necrosis factor (TNF), Caspase-3 (CASP3), Apoptosis regulator Bcl-2 (BCL2). Aflatoxin B_1_ (AFB_1_) strongly interact these proteins with binding energies < −7.5 kcal mol^−1^, confirming their role in liver injury. This docking-based investigations revealed AFB_1_’s disruption of apoptosis pathways enabling rapid, animal-free toxicity risk assessment for food contaminants (Chu and Zi, 2024). Additionally, the possible interactions and toxicity of nanoparticles such as CuO, TiO_2_, ZnO, Mn_2_O_3_, Fe_3_O_4_, Au, Ag, and Fe3O4 with biological targets have also been evaluated using molecular docking (Abdelsattar et al., 2021; Forest, 2022). The reliability of molecular docking predictions is quantitatively assessed using several validation metrics. The root-mean-square deviation (RMSD) is the standard for evaluating pose prediction accuracy; an RMSD of less than 2.0 Å from an experimentally determined co-crystal structure is often considered a successful docking run (Ding et al., 2016). Molecular primarily excels with small, drug-like molecules, but its application to nanoparticles or other non-traditional ligands is often not feasible. The size and multi-point binding modes of nanoparticles fall outside the scope of standard docking algorithms and scoring functions, which are parameterized for atomic-level interactions (Abughalia et al., 2025). Additionally, the accuracy of scoring functions remains a bottleneck, as they can struggle to correctly rank compounds due to approximations in modeling solvation and entropic effects. Recognizing these constraints is crucial for the appropriate application and interpretation of docking studies.

### 4.2 Molecular dynamics simulations and MM-PBSA

**FIGURE 2:**
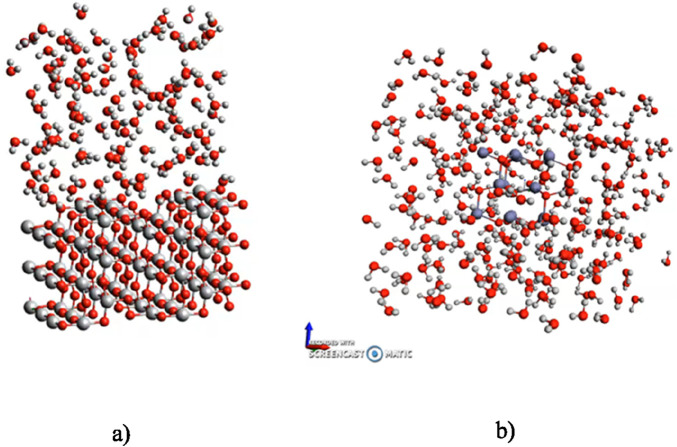
**(a)** 3 × 3 × 2 TiO^2^ slab, exposing the (100) surface. **(b)** 3 × 3 × 2 TiO_2_ nanoparticle, exposing the (100) surface with 108 molecules of water that represent a water density of ∼1 g cm^−3^ in the region of periodic images (Escorihuela Martí, 2019).

MD Simulations use Newton’s mechanics to describe the nuclear motions. Treating atoms and molecules as classical particles gives valuable information about thermodynamics, structures and dynamical properties of the condensed matter from pure liquids to complex biomolecular systems. Molecular dynamics simulations compute motions, so it makes possible to describe position, velocities and changes *versus* time of individual molecules in solids, liquids o gases. With the use of MD simulations coupled with DFTB(DFTB+ simulation package — https://dftbplus.org/index.html), which can incorporate quasi-electronic structure calculations, it may include direct interactions with water and reactivity (Figure 2), we can go a step further in the field of toxicology and calculate physical and chemical properties that can be subsequently used as descriptors (Escorihuela Martí, 2019).

MD simulations play a significant role in structural bioinformatics by helping to understand the behaviour of macromolecules such as proteins and nucleic acids, and how ligands interact with their receptors (Hollingsworth and Dror, 2018; Singh et al., 2018). In food science, MD simulations is a useful technique for studying food processing at the molecular level. It helps optimize flavour and contributes to the development of higher-quality, more functional, nutritionally rich and safer food products (Singh et al., 2018; Smith et al., 2022). Additionally, Molecular Mechanics-Poisson–Boltzmann Surface Area (MM-PBSA) helps utilize trajectory data obtained from MDS to estimate the binding free energy of toxic chemicals with target receptors. Therefore, MDS and MM/PBSA are highly valuable tools for the computational assessment of contaminants present in food (Wang et al., 2018; Jin and Wei, 2024). Bisphenol analogs are frequently detected in food products (Andújar et al., 2019). A recent study demonstrated their interaction with estrogen receptor alpha (ERα). For instance, MDS showed that BPA analogs induce distinct conformational changes in the ligand-binding domain of ERα compared to BPA so that it can be alter its normal behaviour. The findings suggest that computational methods may serve as a reliable approach to assess androgenic and estrogenic activity in materials that come into contact with food (Cavaliere et al., 2020).

To bridge the computational results with toxicological outcomes, the binding free energies (ΔGbind) calculated *via* MM/PBSA provide a quantitative measure of ligand-receptor complex stability. A more negative ΔGbind signifies a more stable interaction, which often correlates with a stronger biological effect (Zare et al., 2024). For example, a high binding affinity for a toxicant towards a key protein, can directly predict the potential for acute toxicity by inhibiting its function (Liu S. et al., 2024; Chalaris et al., 2025). Furthermore, beyond specific protein targets, the principles of molecular interactions explored in these simulations are fundamental for predicting broader ADMET properties. The same forces governing protein-ligand binding also influence a molecule’s passive membrane permeability and bioavailability, key determinants for its absorption and distribution within an organism (Gomes et al., 2023).

### 4.3 QSAR and machine learning models

QSAR (Quantitative Structure-Activity Relationship) (Pradeep et al., 2020; Gajewicz et al., 2015) and machine learning (ML) models (Seal et al., 2025; Hong, 2023) are revolutionizing food toxicology and helps in rapid, cost-effective and predictive assessments of chemical hazards (Pradeep et al., 2020). QSAR and ML are changing how we handle food safety. Instead of waiting to test for toxins after they have caused harm, these tools let us predict risks in advance (Pradeep et al., 2020; Wu et al., 2024). By analyzing a chemical’s structure, applying smart algorithms, and making models easier to interpret, we can quickly flag hazards from microplastics to drug residues in food products (Pradeep et al., 2020; Wu et al., 2024; Yang et al., 2023): The challenges like inconsistent data quality or global regulatory gaps still need teamwork to solve (Bo et al., 2023). Additionally, combining multi-omics data help us stop food toxicity threats before they even emerge (Su et al., 2024; Son et al., 2024). As recent studies show, these methods are becoming vital for initial risk assessments where traditional data is limited, truly reshaping food safety for the future (Honma et al., 2019; Pradeep et al., 2020). Using high-throughput QSAR modelling, researchers screened over 3,000 food-related compounds, ultimately identifying 160 as priority endocrine-disrupting hazards (Yang et al., 2023). Additionally, researchers developed novel QSAR models using cancer-relevant PubChem bioassays and multi-algorithm ML for predicting carcinogenicity. The models successfully prioritized 342 chemicals and identified new potential human carcinogens validated by literature (Chung et al., 2023).

To address key challenges in applying ML approaches to toxicology, we can focus on three critical areas such as adopting standardized characterization protocols to ensure data quality; developing descriptors grounded in mechanistic toxicology to improve interpretability; and finally, aligning entire workflow with OECD-accepted QSAR validation principles for regulatory acceptance (OECD QSAR Toolbox|https://www.oecd.org/en/data/tools/oecd-qsar-toolbox.html).

### 4.4 Network toxicology and pathway analysis

Network and pathway analysis is the key approach that help to visualize the clear picture of biological system. In toxicology, network analysis maps out how toxins engage with the cell’s molecular machinery (Jin et al., 2025), while pathway analysis reveals which cellular processes go off course (Wiklund et al., 2023). By combining these approaches, we can turn complex omics datasets into clear, mechanism-driven insights for assessing food safety paving the way for more precise and predictive evaluations (Zhang S. et al., 2024). Li *et al.* used network toxicology to identify 93 shared molecular targets associated with BPA-induced diabetic cardiomyopathy and related pathways. The study highlights the importance of evaluating BPA substitutes and provides insights to inform regulatory limits on food-contact materials (Li et al., 2025). Lei *et al.* utilized network toxicology to identified 22 core targets from 259 tetracycline targets, revealing mechanisms such as affecting PI3K-Akt/MAPK that justify reevaluating antibiotic residues in food for risk mitigation (Lei et al., 2024; Lei et al., 2024; Lei et al., 2024; Lei et al., 2024).

This network-based approach provides crucial context for molecular-level findings. While docking and QSAR predict specific protein-ligand interactions, network toxicology elucidates the broader biological consequences, showing how a predicted binding event may propagate through a pathway to ultimately evidence as toxicity (Valls-Margarit et al., 2023; Barel and Herwig, 2018).

### 4.5 Physiologically based pharmacokinetic (PBPK) models

**FIGURE 3:**
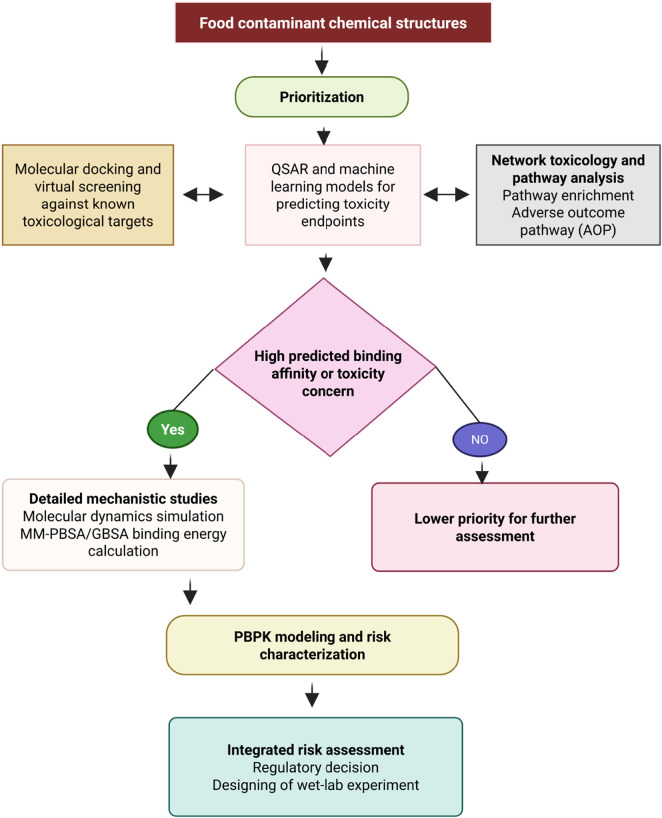
Workflow for computational risk assessment of food contaminants. The pipeline integrates *in silico* methods for hazard prioritization with PBPK modeling. The outcome directs resources toward high-risk compounds for advanced simulation, supporting efficient risk management and hypothesis-driven wet-lab validation.

Physiologically based pharmacokinetic (PBPK) modeling is a powerful predictive toxicology tool that uses a mechanistic framework to describe absorption, distribution, metabolism, and excretion (ADME) of food-borne chemicals (Mi and Lin, 2025; Lipscomb et al., 2012). This approach allows for the estimation of chemical concentrations in human tissues, making it possible to accurately extrapolate data across different species and doses. By accounting for real-world exposure scenarios, PBPK models help to reduce the need for animal testing, inform regulatory decisions, and enhance risk assessments for food contaminants and residues (Lipscomb et al., 2012; Deepika and Kumar, 2023). PBPK models are also key t for predicting food-drug interactions, determining how meals can alter drugs’ behaviours (Riedmaier et al., 2020). For instance, Dede *et al.* used PBPK models to assess human exposure to toxic elements (As, Cd, Cr, Ni, Pb) from home-grown produce. The approach enhances biomonitoring accuracy, supporting human health risk assessment in environmental exposure studies (Riedmaier et al., 2020). Riedmaier *et al.* evaluated PBPK model ‘s ability to predict how food affects the absorption of orally administered drugs using data from 30 compounds. By applying a systematic modeling approach, high to moderate prediction confidence was achieved for most compounds (Riedmaier et al., 2020). Given their importance, several PBPK models have been developed to specifically assess food toxicity and support risk assessment (Li et al., 2017; Ai et al., 2024; Tistaert et al., 2019). Therefore, “*in silico*” approaches have strong potential to elucidate the nature of toxicants in food, and the interconnections between these techniques in supporting risk assessment. Figure 3 depicts an integrative workflow for food contaminant risk assessment, illustrating a pipeline that interconnects computational approaches from chemical structures to system-level prediction. This synergy enhances the robustness of both toxin prioritization and the overall assessment, thereby strengthening the foundation for regulatory decisions.

To further strengthen the applicability of our discussion, we highlight the growing use of PBPK modeling in the risk assessment of food contaminants and animal-derived products. These models are pivotal for predicting tissue residue depletion and establishing extralabel withdrawal intervals for veterinary drugs in food animals, directly ensuring the safety of products like meat, milk, and eggs (Lin et al., 2025). Furthermore, recent advancements are increasingly focused on refining these models to account for human physiological variability using probabilistic methods and Monte Carlo simulations, which provide a more robust estimation of human equivalent doses across diverse populations (Chou and Lin, 2023; Schacht et al., 2024). Finally, the field is being transformed by the integration of machine learning (ML) with PBPK modeling. ML approaches can now predict critical absorption, distribution, metabolism, and excretion (ADME) parameters directly from chemical structure, accelerating the development of robust models for a large number of compounds, even with limited experimental data (Chou and Lin, 2023; Mavroudis et al., 2023). This synergy between mechanistic modeling and data-driven prediction represents a powerful paradigm for high-throughput exposure and risk assessment.

## 5 Experimental validation of computational findings

Computational prediction significantly saves time and funds, enabling the rapid toxicological evaluation of food contaminants. Computational approaches are helpful in prioritizing hazardous contaminants and elucidating their mechanisms of toxic action. However, verifying these computational predictions necessitates rigorous experimental validation. This review discusses integrated computational methodologies, such as molecular docking, molecular dynamics simulations and binding energy calculations, which are used to visualize and predict the interactions between contaminants and human receptors. Experimental techniques, including bioluminescence resonance energy transfer (BRET)-based assays and Western blotting, are employed to understand the subsequent toxic effects (Jeong et al., 2025a; Jeong et al., 2025b). Furthermore, data from *in vivo* animal pharmacokinetic studies are crucial for validating PBPK models (Jobst et al., 2025). ^.^ A recent study employed molecular docking and molecular dynamics to predict interactions of various benzophenones with the androgen receptor, identifying key amino acid residues and binding affinities. These computational predictions were subsequently validated using BRET-based, luciferase assays and Western blot, which showed good agreement and thereby supported the credibility of the computational approach (Jeong et al., 2025a). Jeong et al. (2025b) combined molecular docking and molecular dynamics simulations to explore how methiocarb interacts with estrogen receptor alpha (ERα), revealing key binding residues and predicted binding strengths. These computational insights were confirmed through *in vitro* experiments, demonstrating that methiocarb activates ERα and validating the predictive power of the computational approach (Jeong et al., 2025b). Duan et al. (2025a) utilized a combination of network toxicology and molecular docking to identify 49 common targets linking acrylamide (ACR) exposure to breast cancer (BC). Key genes, including EGFR, FN1, JUN, and COL1A1, were further validated through molecular dynamics simulations, which confirmed binding stability, and immunohistochemistry, which demonstrated their altered expression in tissues. These results suggest a potential mechanistic role for ACR in BC development (Duan et al., 2025a). A study adapted an existing PBPK model to simulate the pharmacokinetics of PFOS and PFOA. The model was validated by comparing its predictions to experimentally measured human data, successfully demonstrating its utility for real-world risk assessment applications (Fàbrega et al., 2014). Therefore, the strong correlation between these computational and experimental approaches not only supports the predictive power of the models but also provides a robust, mechanistically up-to-date foundation for developing targeted mitigation strategies against food contaminants to support health risk assessments.

## 6 Risk assessment and regulatory frameworks

Food regulations are built on a framework that first identifies and assesses hazards, then establishes maximum levels for contaminants, determines their toxicity, monitors products to ensure compliance, and finally evaluates the overall public health impact.

For engineered nanomaterials (ENMs) in food, risk assessment involves evaluating potential hazards and exposures to determine the likelihood and severity of adverse health effects. This process must account for the unique properties of ENMs, their interactions with the body, and potential exposure routes through food. A tiered approach, combining “*in silico”*, *“in vitro”*, and *“in vivo”* studies, is often used to assess ENM safety (Usmani et al., 2024).

Regulatory bodies like the European Food Safety Authority (EFSA) and the U.S. Food and Drug Administration (FDA) play a critical role in this process. EFSA, for example, evaluates the safety of chemicals that could harm humans, animals, or the environment. It has created the OpenFoodTox 2.0 database, an open-source resource detailing critical toxicological endpoints for various substances under European legislation. The FDA identifies and assesses the most significant toxins in food, while the World Health Organization (WHO) works to establish international food safety standards.

Significant efforts have been made to develop specific guidelines for these assessments. In 2011, EFSA published its *Guidance on the risk assessment of the application of nanoscience and nanotechnologies in the food and feed chain* (Committee et al., 2011)^.^ This was followed in 2021 by updated guidance that specifically addressed human and animal health risks. These guidelines emphasize that testing nanomaterials requires consideration of their unique morphological and chemical characteristics, which can alter their biokinetic behavior and toxicological responses compared to their non-nano counterparts. Therefore, applicants must conduct a separate physicochemical characterization and specific risk assessment for each distinct nanomaterial.

In a move towards modernizing risk assessment, EFSA’s Strategy 2027 advocates for the promotion of New Approach Methodologies (NAMs). These methods, based on alternatives to animal experiments, aim to develop cutting-edge techniques and improve the quality and efficiency of hazard risk assessment for chemicals and food-related substances (EFSA Strategy 2027 – Science, safe food, sustainability|EFSA, n. d.)

## 7 Mitigation strategies

Multiple evidence-based approaches exist to mitigate food contamination, including agricultural controls (El Kayal et al., 2024; Periasamy and Gopi, 2023), advanced food processing technologies (Hassan and Zhou, 2018) biological detoxification methods using microbes as well as enzymes and enforced regulatory measures (Hassan and Zhou, 2018). Collectively, these strategies reduce toxin risks to ensure safer food supplies and protect human and environmental health (El Kayal et al., 2024). Onyeaka *et al.* highlight the growing concern of chemical contaminants in food, their sources, and the associated health risks. They emphasize stronger regulations, surveillance, and global best practices to minimize contamination and protect public health, especially in developing nations (Onyeaka et al., 2024).

Agricultural practices are considered the first line of defense in preventing food contamination before it reaches us. Good agricultural practices help minimize biological and chemical hazards, but they require the use of clean irrigation water, healthy soil management, and avoiding harmful chemical pesticides. On the animal food side, it is important to maintain animal health through proper vaccinations and ensure that animal feed is free from contaminants. Training farmers is also essential to support these practices (Lepper et al., 2017). Advanced food processing approaches such as Pulsed Electric Fields (PEF) (Ghoshal, 2023), High Pressure Processing (HPP) (Koutsoumanis et al., 2022), and Cold Plasma (Harikrishna et al., 2023) play a significant role in reducing contaminants and inactivating pathogens in food products. Therefore, these technologies hold immense potential to be employed more frequently for mitigating toxins in food (Nunes et al., 2021).

Biological detoxification using microbes such as lactic acid bacteria (Średnicka et al., 2021; Muhialdin et al., 2020) and enzymes like laccases, reductases, oxidases, and peroxidases (Liu M. et al., 2024) helps to degrade and transform contaminants such as mycotoxins, pesticides, and allergens. These methods provide targeted, eco-friendly alternatives to chemical treatments, enhancing food safety by mitigating harmful substances (Abraham et al., 2022; Średnicka et al., 2021; Karlovsky et al., 2016).

Additionally, regulatory measures play a crucial role in the mitigation of contaminants in food (Food Chemical Safety|FDA, 2025), (Yu, 2024), Regional comparisons of regulatory systems reveal distinct differences shaped by variations in resources, infrastructure, and enforcement capacity (Duan et al., 2025a). Therefore, international, regional, and national regulatory frameworks can help to establish multilayered controls to mitigate food contamination risks and protect consumers globally (Duan et al., 2025b). Predictive modeling supports mitigation efforts by enabling the *in silico* design of safer materials, such as simulating polymer coatings to minimize nanoparticle release. QSAR models can proactively identify structural features that contribute to toxicity, helping researchers design or select chemical alternatives with reduced environmental impact (Shan et al., 2024).

The standardisation of the experimental tests is required to validate the results of modelling studies. It is necessary that this standardisation works together with computational and experimental methodologies to gain future insight into risk assessment and other challenges. For instance, the database https://data.enanomapper.net/ where many projects from European Union have been introduced their experimental data of toxicity characterisation for risk assessment. The use of *in silico* methods and advanced machine learning techniques to obtain NP descriptors reals for experimental comparison may become the next standard procedure to accelerate the evaluation of toxicity.

## 8 Conclusion

This review has elucidated the critical role of computational methods in analyzing the toxicity mechanisms of food contaminants. The advancing age of techniques like molecular docking, MD simulations, QSAR, and PBPK modeling now positions them for robust use in regulatory screening and prioritization. However, critical gaps remain. For nanoparticles, these include a shortage of high-quality reference data for model validation and the limited development of nanomaterial-specific models. The most promising frontier lies in the planned integration of machine learning with established mechanistic models, building hybrid frameworks that are both predictive and interpretable. This evolving computational toolkit is the foundation of a practical “safety-by-design” paradigm, enabling the proactive identification of hazards before products enter the market. To realize this potential, a intensive effort towards international harmonization of regulatory standards is essential. Through supporting these advanced, ethical tools, we can not only safeguard the global food supply but also foster responsible innovation, ensuring that public health protection keeps pace with technological advancement.

## References

[B1] Abdelsattar A. S. Dawoud A. Helal M. A. (2021). Interaction of nanoparticles with biological macromolecules: a review of molecular docking studies. Nanotoxicology 15, 66–95. 10.1080/17435390.2020.1842537 33283572

[B2] Abraham M. J. Murtola T. Schulz R. Páll S. Smith J. C. Hess B. (2015). GROMACS: high performance molecular simulations through multi-level parallelism from laptops to supercomputers. SoftwareX 1–2, 19–25. 10.1016/J.SOFTX.2015.06.001 1

[B3] Abraham N. Chan E. T. S. Zhou T. Seah S. Y. K. (2022). Microbial detoxification of mycotoxins in food. Front. Microbiol. 13, 957148. 10.3389/FMICB.2022.957148/XML 36504774 PMC9726736

[B4] Abram S. L. Tavernaro I. Johnston L. J. Zou S. Resch-Genger U. (2025). Nanoscale reference and test materials for the validation of characterization methods for engineered nanomaterials — current state, limitations, and needs. Anal. Bioanal. Chem. 417, 2405–2425. 10.1007/S00216-024-05719-6/TABLES/2 39754617 PMC12003566

[B5] Abughalia A. Flynn M. Clarke P. F. A. Fayne D. Gobbo O. L. (2025). The use of computational approaches to design nanodelivery systems. Nanomaterials 15, 1354. 10.3390/NANO15171354 40938032 PMC12430099

[B6] Agnihotry S. Pathak R. K. Srivastav A. Shukla P. K. Gautam B. (2020). Molecular docking and structure-based drug design. Computer-Aided Drug Des., 115–131. 10.1007/978-981-15-6815-2_6

[B7] Ahn C. Jeung E. B. (2023). Endocrine-disrupting chemicals and disease endpoints. Int. J. Mol. Sci. 24, 5342. 10.3390/IJMS24065342 36982431 PMC10049097

[B8] Ai J. Gao Y. Yang F. Zhao Z. Dong J. Wang J. (2024). Corrigendum: development and application of a physiologically-based pharmacokinetic model for ractopamine in goats. Front. Vet. Sci. 11, 1523431. 10.3389/FVETS.2024.1523431/BIBTEX 39641095 PMC11617936

[B9] Ameta S. K. Rai A. K. Hiran D. Ameta R. Ameta S. C. (2020). “Use of nanomaterials in food science,” in Biogenic nano-particles and their use in agro-ecosystems (Singapore: Springer Singapore), 457–488. 10.1007/978-981-15-2985-6_24

[B10] Andújar N. Gálvez-Ontiveros Y. Zafra-Gómez A. Rodrigo L. Álvarez-Cubero M. J. Aguilera M. (2019). Bisphenol A analogues in food and their hormonal and obesogenic effects: a review. Nutrients 11, 1–18. 10.3390/nu11092136 31500194 PMC6769843

[B11] Anjum A. Garg R. Kashif M. Eddy N. O. (2023). Nano-scale innovations in packaging: properties, types, and applications of nanomaterials for the future. Food Chem. Adv. 3, 100560. 10.1016/J.FOCHA.2023.100560

[B12] Ashfaq A. Khursheed N. Fatima S. Anjum Z. Younis K. (2022). Application of nanotechnology in food packaging: Pros and Cons. J. Agric. Food Res. 7, 100270. 10.1016/J.JAFR.2022.100270

[B13] Attarilar S. Yang J. Ebrahimi M. Wang Q. Liu J. Tang Y. (2020). The toxicity phenomenon and the related occurrence in metal and metal oxide nanoparticles: a brief review from the biomedical perspective. Front. Bioeng. Biotechnol. 8. 822. 10.3389/fbioe.2020.00822 32766232 PMC7380248

[B14] Barel G. Herwig R. (2018). Network and pathway analysis of toxicogenomics data. Front. Genet. 9, 408961. 10.3389/FGENE.2018.00484/BIBTEX 30405693 PMC6204403

[B15] Bastos V. Ferreira de Oliveira J. Brown D. Jonhston H. Malheiro E. Daniel-da-Silva A. (2016). The influence of citrate or PEG coating on silver nanoparticle toxicity to a human keratinocyte cell line citation for published version:the influence of citrate or PEG coating on silver nanoparticle toxicity to a human keratinocyte cell line. Toxicol. Lett. 249, 29–41. 10.1016/j.toxlet.2016.03.005 27021274

[B16] Batir-Marin D. Boev M. Cioanca O. Lungu I. I. Marin G. A. Burlec A. F. (2025). Exploring oxidative stress mechanisms of nanoparticles using zebrafish (*Danio rerio*): toxicological and pharmaceutical insights. Antioxidants 14, 489. 10.3390/antiox14040489 40298867 PMC12024358

[B17] Bisphenol (n.d.). Bisphenol A in food is a health risk|EFSA. Available online at: https://www.efsa.europa.eu/en/news/bisphenol-food-health-risk (Accessed October 02, 2025).

[B18] Biswas R. Alam M. Sarkar A. Haque M. I. Hasan M. M. Hoque M. (2022). Application of nanotechnology in food: processing, preservation, packaging and safety assessment. Heliyon 8, e11795. 10.1016/J.HELIYON.2022.E11795 36444247 PMC9699984

[B19] Bo T. Lin Y. Han J. Hao Z. Liu J. (2023). Machine learning-assisted data filtering and QSAR models for prediction of chemical acute toxicity on rat and mouse. J. Hazard Mater 452, 131344. 10.1016/j.jhazmat.2023.131344 37027914

[B20] Cavaliere F. Lorenzetti S. Cozzini P. (2020). Molecular modelling methods in food safety: bisphenols as case study. Food Chem. Toxicol. 137, 111116. 10.1016/j.fct.2020.111116 31931072

[B21] Çetin Y. A. Martorell B. Serratosa F. Calatayud M. (2024). Adsorption of guanine on oxygen-deficient TiO_2_ surface: a combined MD-DFTB/DFT strategy. ACS Omega 9, 39043–39050. 10.1021/acsomega.4c05806 39310186 PMC11411692

[B22] Chalaris M. Koufou A. Anastasiou S. Roupas P. A. Nikolaou G. (2025). Exploring the physicochemical and toxicological study of G-Series and A-Series agents combining molecular dynamics and quantitative structure–activity relationship. ChemEngineering 9, 91. 10.3390/CHEMENGINEERING9040091

[B23] Chen W. Deng Y. Russell E. Wu Y. Abel R. Wang L. (2018). Accurate calculation of relative binding free energies between ligands with different net charges. J. Chem. Theory Comput. 14, 6346–6358. 10.1021/ACS.JCTC.8B00825/ASSET/IMAGES/MEDIUM/CT-2018-00825E_0008.GIF 30375870

[B24] Chen B. Li M. Gu Y. Lou W. Huang S. Mao F. (2025). Unraveling the carcinogenic mechanisms of food contaminants: an integrated *in silico* framework combining network toxicology, machine learning, and molecular docking. J. Food Sci. 90, e70697. 10.1111/1750-3841.70697 41246859 PMC12621292

[B25] Cherian J. J. Taube A. G. McGibbon R. T. Angelikopoulos P. Blanc G. Snarski M. (2020). Efficient hyperparameter optimization by way of PAC-Bayes bound minimization. Available online at: http://arxiv.org/abs/2008.06431 (Accessed July 21, 2025).

[B26] Chou W. C. Lin Z. (2023). Machine learning and artificial intelligence in physiologically based pharmacokinetic modeling. Toxicol. Sci. 191, 1–14. 10.1093/TOXSCI/KFAC101 36156156 PMC9887681

[B27] Chu Z. Y. Zi X. J. (2024). Network toxicology and molecular docking for the toxicity analysis of food contaminants: a case of Aflatoxin B1. Food Chem. Toxicol. 188, 114687. 10.1016/j.fct.2024.114687 38663764

[B28] Chung E. Russo D. P. Ciallella H. L. Wang Y. T. Wu M. Aleksunes L. M. (2023). Data-driven quantitative structure-activity relationship modeling for human carcinogenicity by chronic oral exposure. Environ. Sci. Technol. 57, 6573–6588. 10.1021/ACS.EST.3C00648/ASSET/IMAGES/MEDIUM/ES3C00648_0010.GIF 37040559 PMC10134506

[B29] Committee S. Barlow S. Chesson A. Flynn A. Hardy A. Silano V. (2011). Guidance on the risk assessment of the application of nanoscience and nanotechnologies in the food and feed chain. EFSA J. 9.

[B30] Corcuera L. A. Vettorazzi A. Arbillaga L. Pérez N. Gil A. G. Azqueta A. (2015). Genotoxicity of Aflatoxin B1 and Ochratoxin A after simultaneous application of the *in vivo* micronucleus and comet assay. Food Chem. Toxicol. 76, 116–124. 10.1016/J.FCT.2014.12.003 25530104

[B31] Cytoscape (n.d.). An open source platform for complex network analysis and visualization. Available online at: https://cytoscape.org/(Accessed July 21, 2025).

[B32] Dai C. Tian E. Hao Z. Tang S. Wang Z. Sharma G. (2022). Aflatoxin B1 toxicity and protective effects of curcumin: molecular mechanisms and clinical implications. Antioxidants 11, 2031. 10.3390/ANTIOX11102031 36290754 PMC9598162

[B33] Dede E. Tindall M. J. Cherrie J. W. Hankin S. Collins C. (2018). Physiologically-based pharmacokinetic and toxicokinetic models for estimating human exposure to five toxic elements through oral ingestion. Environ. Toxicol. Pharmacol. 57, 104–114. 10.1016/J.ETAP.2017.12.003 29253785

[B34] Deepika D. Kumar V. (2023). The role of “physiologically based pharmacokinetic model (PBPK)” new approach methodology (NAM) in pharmaceuticals and environmental chemical risk assessment. Int. J. Environ. Res. Public Health 20, 3473. 10.3390/IJERPH20043473 36834167 PMC9966583

[B35] Desmond (n.d.). Schrödinger life science. Available online at: https://www.schrodinger.com/platform/products/desmond/ (Accessed July 21, 2025).

[B36] DFTB (n.d.). DFTB+ simulation package — DFTB+. Available online at: https://dftbplus.org/ (Accessed July 22, 2025).

[B37] Dimitrijević M. Stanković M. Nikolić J. Mitić V. Stankov Jovanović V. Stojanović G. (2023). The effect of arsenic, cadmium, mercury, and lead on the genotoxic activity of Boletaceae family mushrooms present in Serbia. J. Toxicol. Environ. Health A 86, 23–35. 10.1080/15287394.2022.2150992 36445018

[B38] Ding Y. Fang Y. Moreno J. Ramanujam J. Jarrell M. Brylinski M. (2016). Assessing the similarity of ligand binding conformations with the contact mode score. Comput. Biol. Chem. 64, 403–413. 10.1016/J.COMPBIOLCHEM.2016.08.007 27620381 PMC5159245

[B39] Duan K. Pang G. Duan Y. Onyeaka H. Krebs J. (2025a). Current research development on food contaminants, future risks, regulatory regime and detection technologies: a systematic literature review. J. Environ. Manage. 381, 125246. 10.1016/J.JENVMAN.2025.125246 40199209

[B40] Duan Z. Yu C. Yang W. Wang W. Zhang Q. Ruan Q. (2025b). Network toxicology and molecular docking reveal the potential link between acrylamide exposure and breast cancer. Sci. Rep. 15, 1–13. 10.1038/S41598-025-06964-0 40593011 PMC12217316

[B199] Eberhardt J. Santos-Martins D. Tillack A. F. Forli S. (2021). AutoDock vina 1.2.0: new docking methods, expanded force field, and python bindings. J. Chem. Inf. Model. 61 (8), 3891–3898. 10.1021/acs.jcim.1c00203 34278794 PMC10683950

[B41] EFSAa (2021). Chemical contaminants in food and feed|EFSA. Available online at: https://www.efsa.europa.eu/en/topics/topic/chemical-contaminants-food-feed (Accessed July 11, 2025).

[B42] EFSA EFSA strategy 2027 – science, safe food, sustainability|EFSA (n.d.). Available online at: https://www.efsa.europa.eu/en/corporate-pubs/efsa-strategy-2027-science-safe-food-sustainability (Accessed July 08, 2025).

[B43] Eisenbrand G. (2020a). Correction to: revisiting the evidence for genotoxicity of acrylamide (AA), key to risk assessment of dietary AA exposure. Arch. Toxicol. 94, 3935. 10.1007/S00204-020-02893-1 32935137 PMC7852880

[B44] Eisenbrand G. (2020b). Correction to: Revisiting the evidence for genotoxicity of acrylamide (AA), key to risk assessment of dietary AA exposure (Archives of Toxicology, (2020), 94, 9, (2939-2950), 10.1007/s00204-020-02794-3). Arch. Toxicol. 94, 3935. 10.1007/S00204-020-02893-1/METRICS 32935137 PMC7852880

[B45] El Kayal W. Darwiche L. Farhat Y. A. Hdeib M. AlJardaly R. Shbaro M. (2024). Agricultural mitigation strategies to reduce the impact of romaine lettuce contamination. Plants 13, 2460. 10.3390/PLANTS13172460/S1 39273944 PMC11396837

[B46] Escher S. E. Partosch F. Konzok S. Jennings P. Luijten M. Kienhuis A. (n.d.). External scientific report development of a roadmap for action on new approach methodologies in risk assessment roadmap for action on new approach methodologies. 19 (6). 10.2903/sp.efsa.2022.EN-7341

[B47] Escorihuela L. Fernández A. Rallo R. Martorell B. (2018a). Molecular dynamics simulations of zinc oxide solubility: from bulk down to nanoparticles. Food Chem. Toxicol. 112, 518–525. 10.1016/j.fct.2017.07.038 28736190

[B48] Escorihuela L. Martorell B. Rallo R. Fernández A. (2018b). Toward computational and experimental characterisation for risk assessment of metal oxide nanoparticles. Environ. Sci. Nano 5, 2241–2251. 10.1039/C8EN00389K

[B49] Escorihuela Martí L. (2019). Computational characterisation of metal oxide nanoparticles for hazard screening and risk assessment. TDX Tesis Doctorals en Xarxa. Available online at: http://www.tdx.cat/handle/10803/669615 (Accessed December 23, 2021).

[B50] Espitia P. J. P. De Fátima Ferreira Soares N. Teófilo R. F. Dos Reis Coimbra J. S. Vitor D. M. Batista R. A. (2013). Physical–mechanical and antimicrobial properties of nanocomposite films with pediocin and ZnO nanoparticles. Carbohydr. Polym. 94, 199–208. 10.1016/J.CARBPOL.2013.01.003 23544529

[B51] Fàbrega F. Kumar V. Schuhmacher M. Domingo J. L. Nadal M. (2014). PBPK modeling for PFOS and PFOA: validation with human experimental data. Toxicol. Lett. 230, 244–251. 10.1016/J.TOXLET.2014.01.007 24440341

[B52] Faria M. Navas J. M. Soares A. M. V. M. Barata C. (2015). Corrigendum to “Oxidative stress effects of titanium dioxide nanoparticle aggregates in zebrafish” [Sci. Total Environ. Volumes 470–471, 1 February 2014, Pages 379–389]. Sci. Total Environ. 512-513, 686. 10.1016/J.SCITOTENV.2015.02.006 28807471

[B53] Flores-López L. Z. Espinoza-Gómez H. Somanathan R. (2019). Silver nanoparticles: electron transfer, reactive oxygen species, oxidative stress, beneficial and toxicological effects. Mini review. J. Appl. Toxicol. 39, 16–26. 10.1002/JAT.3654 29943411

[B54] Food Chemical Safety FDA (2025). Available online at: https://www.fda.gov/food/food-ingredients-packaging/food-chemical-safety (Accessed July 21, 2025).

[B55] Food Safety (2025). Food safety. Available online at: https://www.who.int/news-room/fact-sheets/detail/food-safety (Accessed November 28, 2025).

[B56] Forest V. (2022). Experimental and computational Nanotoxicology— complementary approaches for nanomaterial hazard assessment. Nanomaterials 12, 1346. 10.3390/nano12081346 35458054 PMC9031966

[B57] Friesner R. A. Banks J. L. Murphy R. B. Halgren T. A. Klicic J. J. Mainz D. T. (2004). Glide: a new approach for rapid, accurate docking and scoring. 1. Method and assessment of docking accuracy. J. Med. Chem. 47, 1739–1749. 10.1021/JM0306430/SUPPL_FILE/JM0306430_S.PDF 15027865

[B58] Fuciños C. Fuciños P. Estévez N. Pastrana L. M. Vicente A. A. Rúa M. L. (2019). One-step chromatographic method to purify α-lactalbumin from whey for nanotube synthesis purposes. Food Chem. 275, 480–488. 10.1016/J.FOODCHEM.2018.09.144 30724223

[B59] Gajewicz A. Schaeublin N. Rasulev B. Hussain S. Leszczynska D. Puzyn T. (2015). Towards understanding mechanisms governing cytotoxicity of metal oxides nanoparticles: hints from nano-QSAR studies towards understanding mechanisms governing cytotoxicity of metal oxides nanoparticles: hints from nano-QSAR studies. 5390. 313–325 10.3109/17435390.2014.930195 24983896

[B60] Genheden S. Ryde U. (2015). The MM/PBSA and MM/GBSA methods to estimate ligand-binding affinities. Expert Opin. Drug Discov. 10, 449–461. 10.1517/17460441.2015.1032936 25835573 PMC4487606

[B61] Ghoshal G. (2023). Comprehensive review on pulsed electric field in food preservation: gaps in current studies for potential future research. Heliyon 9, e17532. 10.1016/J.HELIYON.2023.E17532/ASSET/DDAC4C6D-345A-49EC-8372-C182BB79AB30/MAIN.ASSETS/GR3.JPG 37408918 PMC10318501

[B62] Gokso̷yr S. Ø. Yadetie F. Johansen C. T. Jacobsen R. G. Lille-Lango̷y R. Gokso̷yr A. (2024). Interaction of bisphenol A and its analogs with estrogen and androgen receptor from Atlantic cod (*Gadus morhua*). Environ. Sci. Technol. 58, 14098–14109. 10.1021/ACS.EST.4C01500/ASSET/IMAGES/LARGE/ES4C01500_0005.JPEG 39087390 PMC11325555

[B63] Gomes A. M. M. Costa P. J. Machuqueiro M. (2023). Recent advances on molecular dynamics-based techniques to address drug membrane permeability with atomistic detail. BBA Adv. 4, 100099. 10.1016/J.BBADVA.2023.100099 37675199 PMC10477461

[B64] Gosslau A. (2016). Assessment of food toxicology. Food Sci. Hum. Wellness 5, 103–115. 10.1016/j.fshw.2016.05.003

[B65] Gupta R. K. Guha P. Srivastav P. P. (2024). Investigating the toxicological effects of nanomaterials in food packaging associated with human health and the environment. J. Hazard. Mater. Lett. 5, 100125. 10.1016/j.hazl.2024.100125

[B66] Harikrishna S. Anil P. P. Shams R. Dash K. K. (2023). Cold plasma as an emerging nonthermal technology for food processing: a comprehensive review. J. Agric. Food Res. 14, 100747. 10.1016/J.JAFR.2023.100747

[B67] Hassan Y. I. Zhou T. (2018). Promising detoxification strategies to mitigate mycotoxins in food and feed. Toxins 10, 116. 10.3390/TOXINS10030116 29522477 PMC5869404

[B68] Heikkinen T. Küblbeck J. Rysä J. (2025). Metabolic disruption by mycotoxins: focus on metabolic endpoints steatosis, adipogenesis and glucose metabolism *in vivo* and *in vitro* . Arch. Toxicol. 99, 1749–1767. 10.1007/S00204-025-03957-W 39923202 PMC12085319

[B69] Hilty F. M. Knijnenburg J. T. N. Teleki A. Krumeich F. Hurrell R. F. Pratsinis S. E. (2011). Incorporation of Mg and Ca into nanostructured Fe2O3 improves Fe solubility in dilute acid and sensory characteristics in foods. J. Food Sci. 76, N2–N10. 10.1111/J.1750-3841.2010.01885.X;WGROUP:STRING:PUBLICATION 21535701

[B70] Hollingsworth S. A. Dror R. O. (2018). Molecular dynamics simulation for all. Neuron 99, 1129–1143. 10.1016/j.neuron.2018.08.011 30236283 PMC6209097

[B71] Hong H. (2023). Machine learning and deep learning in computational toxicology. Cham: Springer. 10.1007/978-3-031-20730-3

[B72] Honma M. Kitazawa A. Cayley A. Williams R. V. Barber C. Hanser T. (2019). Improvement of quantitative structure–activity relationship (QSAR) tools for predicting ames mutagenicity: outcomes of the Ames/QSAR International Challenge Project. Mutagenesis 34, 3–16. 10.1093/MUTAGE/GEY031 30357358 PMC6402315

[B73] Hou T. Guo S. Xu X. (2002). Predictions of binding of a diverse set of ligands to gelatinase-A by a combination of molecular dynamics and continuum solvent models. J. Phys. Chem. B 106, 5527–5535. 10.1021/JP015516Z/ASSET/IMAGES/LARGE/JP015516ZF00006.JPEG

[B74] Hou T. Chen K. McLaughlin W. A. Lu B. Wang W. (2006). Computational analysis and prediction of the binding motif and protein interacting partners of the abl SH3 domain. PLoS Comput. Biol. 2, e1. 10.1371/JOURNAL.PCBI.0020001 16446784 PMC1356089

[B75] Hou T. Wang J. Li Y. Wang W. (2011). Assessing the performance of the MM/PBSA and MM/GBSA methods. 1. The accuracy of binding free energy calculations based on molecular dynamics simulations. J. Chem. Inf. Model 51, 69–82. 10.1021/CI100275A/SUPPL_FILE/CI100275A_SI_001 21117705 PMC3029230

[B76] Huang Y. Li P. Zhao R. Zhao L. Liu J. Peng S. (2022). Silica nanoparticles: biomedical applications and toxicity. Biomed. and Pharmacother. 151, 113053. 10.1016/J.BIOPHA.2022.113053 35594717

[B77] Iori S. Lahtela-Kakkonen M. D’Onofrio C. Maietti F. Mucignat G. Bardhi A. (2024). New insights into aflatoxin B1 mechanistic toxicology in cattle liver: an integrated approach using molecular docking and biological evaluation in CYP1A1 and CYP3A74 knockout BFH12 cell lines. Archives Toxicol. 98 (9), 3097–3108. 10.1007/S00204-024-03799-Y PMC11324698 38834875

[B78] Isoherranen N. (2025). Physiologically based pharmacokinetic modeling of small molecules: how much progress have we made? Drug Metabolism Dispos. 53, 100013. 10.1124/DMD.123.000960 39884807 PMC13021368

[B79] Jadhav V. Gurgude R. Sawant R. (2025). The dawn of animal-free safety: a comprehensive review of new approach methodologies in 21st century toxicology. Next Res. 2, 100986. 10.1016/J.NEXRES.2025.100986

[B80] Jeong D. H. Pathak R. K. Jung D. W. Kim J. M. Lee H. S. (2025a). Exploring the relationship between the androgen receptor and structural configuration of benzophenones: a combination of computational analysis and laboratory models. Environ. Int. 202, 109632. 10.1016/J.ENVINT.2025.109632 40633518

[B81] Jeong D. H. Pathak R. K. Lee E. Kim J. M. Lee H. S. (2025b). Integrative *in silico* and *in vitro* approach for clarifying mode of action to activate estrogen receptor alpha and lipid accumulation by methiocarb. Ecotoxicol. Environ. Saf. 303, 118799. 10.1016/J.ECOENV.2025.118799 40753772

[B82] Jin Z. Wei Z. (2024). Molecular simulation for food protein–ligand interactions: a comprehensive review on principles, current applications, and emerging trends. Compr. Rev. Food Sci. Food Saf. 23, 1–29. 10.1111/1541-4337.13280 38284571

[B83] Jin J. Xue Y. Tian L. (2025). Network toxicological analysis of sodium dehydroacetate in food safety. Food Chem. Toxicol. 201, 115469. 10.1016/J.FCT.2025.115469 40274010

[B84] Jobst M. Guerreiro I. Payros D. Oswald I. P. Fernandes A. S. Warth B. (2025). New approach methodologies for risk assessment of urinary occurring toxicants. Ecotoxicol. Environ. Saf. 302, 118649. 10.1016/J.ECOENV.2025.118649 40749389

[B85] Kar P. Oriola A. O. Oyedeji A. O. (2024). Molecular docking approach for biological interaction of green synthesized nanoparticles. Molecules 29, 1–16. 10.3390/molecules29112428 38893302 PMC11173450

[B86] Karlovsky P. Suman M. Berthiller F. De Meester J. Eisenbrand G. Perrin I. (2016). Impact of food processing and detoxification treatments on mycotoxin contamination. Mycotoxin Res. 32 (4), 179–205. 10.1007/S12550-016-0257-7 27554261 PMC5063913

[B87] Keval R. Tejas G. (2022). Basics, types and applications of molecular docking: a review. IP Int. J. Compr. Adv. Pharmacol. 7, 12–16. 10.18231/J.IJCAAP.2022.003

[B88] Kopp B. Vignard J. Mirey G. Fessard V. Zalko D. Le Hgarat L. (2018). Genotoxicity and mutagenicity assessment of food contaminant mixtures present in the French diet. Environ. Mol. Mutagen 59, 742–754. 10.1002/EM.22214 30230031

[B89] Koutsoumanis K. Alvarez-Ordóñez A. Bolton D. Bover-Cid S. Chemaly M. Davies R. (2022). The efficacy and safety of high-pressure processing of food. EFSA J. 20, e07128. 10.2903/J.EFSA.2022.7128 35281651 PMC8902661

[B90] Krewski D. Acosta D. Andersen M. Anderson H. Bailar J. C. Boekelheide K. (2010). Toxicity testing in the 21st century: a vision and a strategy. J. Toxicol. Environ. Health B Crit. Rev. 13, 51–138. 10.1080/10937404.2010.483176 20574894 PMC4410863

[B91] Kuepfer L. Niederalt C. Wendl T. Schlender J. F. Willmann S. Lippert J. (2016). Applied concepts in PBPK modeling: how to build a PBPK/PD model, CPT Pharmacometrics Syst. Pharmacol. 5, 516–531. 10.1002/PSP4.12134/FULL 27653238 PMC5080648

[B92] Kuppusamy S. Meivelu M. Praburaman L. Mujahid Alam M. Al-Sehemi A. G. K A. (2024). Integrating AI in food contaminant analysis: enhancing quality and environmental protection. J. Hazard. Mater. Adv. 16, 100509. 10.1016/J.HAZADV.2024.100509

[B93] Kutsarova S. Schultz T. W. Chapkanov A. Cherkezova D. Mehmed A. Stoeva S. (2021). The QSAR Toolbox automated read-across workflow for predicting acute oral toxicity: II. Verification and validation. Comput. Toxicol. 20, 100194. 10.1016/J.COMTOX.2021.100194 34293429

[B94] Lei H. Wu Y. Ma W. Yao J. Zhang P. Tian Y. (2024). Network toxicology and molecular docking analysis of tetracycline-induced acute pancreatitis: unveiling core mechanisms and targets. Toxics 12, 929. 10.3390/TOXICS12120929/S1 39771144 PMC11679059

[B95] Lepper J. A. Schneider K. R. Goodrich Schneider R. M. Sreedharan A. (2017). Food safety on the farm: good agricultural practices and good handling practices – worker health and hygiene. EDIS 2017. 10.32473/EDIS-FS158-2017

[B96] Li L. Wang Q. Zhang Y. Niu Y. Yao X. Liu H. (2015). The molecular mechanism of bisphenol A (BPA) as an endocrine disruptor by interacting with nuclear receptors: insights from molecular dynamics (MD) simulations. PLoS One 10, e0120330. 10.1371/JOURNAL.PONE.0120330 25799048 PMC4370859

[B97] Li X. Shi L. Tang X. Wang Q. Zhou L. Song W. (2017). Mechanistic prediction of food effects for Compound A tablet using PBPK model. Saudi J. Biol. Sci. 24, 603–609. 10.1016/J.SJBS.2017.01.032 28386186 PMC5372427

[B98] Li B. Zhao X. Ding Y. Zhang Y. (2025). Network toxicology and molecular docking to investigate the mechanism of bisphenol A toxicity in human diabetic cardiomyopathy. Ecotoxicol. Environ. Saf. 299, 118301. 10.1016/j.ecoenv.2025.118301 40393322

[B99] Liang Y. Jiang Q. Gong Y. Yu Y. Zou H. Zhao J. (2023). *In vitro* and *in silico* assessment of endocrine disrupting effects of food contaminants through pregnane X receptor. Food Chem. Toxicol. 175, 113711. 10.1016/J.FCT.2023.113711 36893891

[B100] Lin Z. Wu X. Mi K. Baynes R. E. Tell L. A. Riviere J. E. (2025). Applications of PBPK models to predict tissue residues and extralabel withdrawal times of drugs in food animals: perspectives from the Food Animal Residue Avoidance Databank (FARAD) Program. AAPS J. 27 (6), 153. 10.1208/S12248-025-01149-Z 41053499

[B101] Lipscomb J. C. Haddad S. Poet T. Krishnan K. (2012). Physiologically-based pharmacokinetic (PBPK) models in toxicity testing and risk assessment. Adv. Exp. Med. Biol. 745, 76–95. 10.1007/978-1-4614-3055-1_6 22437814

[B102] Liu R. Zhang H. Y. Ji Z. X. Rallo R. Xia T. Chang C. H. (2013). Development of structure–activity relationship for metal oxide nanoparticles. Nanoscale 5, 5644–5653. 10.1039/C3NR01533E 23689214

[B103] Liu M. Zhang X. Luan H. Zhang Y. Xu W. Feng W. (2024a). Bioenzymatic detoxification of mycotoxins. Front. Microbiol. 15, 1434987. 10.3389/FMICB.2024.1434987/XML 39091297 PMC11291262

[B104] Liu S. Dong Y. Chen Y. Yang Y. Ni H. Zou X. (2024b). A green approach coupled with molecular dynamics simulations and toxicity assays to infer the mode of action for organophosphate esters. Sci. Total Environ. 955, 177147. 10.1016/J.SCITOTENV.2024.177147 39442719

[B105] Liu L. Xu Y. Ma Y. Duan F. Wang C. Feng J. (2025). Fate of polystyrene micro- and nanoplastics in zebrafish liver cells: influence of protein corona on transport, oxidative stress, and glycolipid metabolism. J. Hazard Mater 489, 137596. 10.1016/j.jhazmat.2025.137596 39952126

[B106] Maden S. F. Sezer S. Acuner S. E. Maden S. F. Sezer S. Acuner S. E. (2022). Fundamentals of molecular docking and comparative analysis of Protein–small-molecule docking approaches. 2. 10.5772/INTECHOPEN.105815

[B107] Mafe A. N. Büsselberg D. (2024). Mycotoxins in food: cancer risks and strategies for control. Foods 13, 3502. 10.3390/FOODS13213502 39517285 PMC11545588

[B108] Mallia J. D. O. Galea R. Nag R. Cummins E. Gatt R. Valdramidis V. (2022). Nanoparticle food applications and their toxicity: current trends and needs in risk assessment strategies. J. Food Prot. 85, 355–372. 10.4315/JFP-21-184 34614149

[B109] Martirosyan A. Schneider Y. J. (2014). Engineered nanomaterials in food: implications for food safety and consumer health. Int. J. Environ. Res. Public Health 11, 5720–5750. 10.3390/ijerph110605720 24879486 PMC4078545

[B110] Mavroudis P. D. Teutonico D. Abos A. Pillai N. (2023). Application of machine learning in combination with mechanistic modeling to predict plasma exposure of small molecules. Front. Syst. Biol. 3, 1180948. 10.3389/FSYSB.2023.1180948/BIBTEX 40809487 PMC12342024

[B111] Mengeling B. J. Ettayapuram Ramaprasad A. S. Smith M. T. Turkieh D. Kleinstreuer N. C. Mansouri K. (2025). An *in silico* to *in vivo* approach identifies retinoid-X receptor activating tert-butylphenols used in food contact materials. Sci. Rep. 15, 26102. 10.1038/s41598-025-09244-z 40681575 PMC12274580

[B112] Mi K. Lin Z. (2025). Chemical risk assessment in food animals *via* physiologically based pharmacokinetic modeling – part II: environmental pollutants on animal and human health assessments. Environ. Int. 198, 109372. 10.1016/j.envint.2025.109372 40106874

[B113] Mi K. Wu X. Lin Z. (2025). Chemical risk assessment in food animals *via* physiologically based pharmacokinetic modeling − Part I: veterinary drugs on human food safety assessment. Environ. Int. 197, 109339. 10.1016/J.ENVINT.2025.109339 39986004

[B114] Mikolajczyk A. Annapoorani V. K. Bahl A. Blekos K. Burk J. Çetin Y. A. (2023). A computational view on nanomaterial intrinsic and extrinsic features for nanosafety and sustainability. Mater. TodayKidlingt. 67, 344–370. 10.1016/j.mattod.2023.05.029

[B115] Misra S. K. Dybowska A. Berhanu D. Luoma S. N. Valsami-Jones E. (2012). The complexity of nanoparticle dissolution and its importance in nanotoxicological studies. Sci. Total Environ. 438, 225–232. 10.1016/j.scitotenv.2012.08.066 23000548

[B116] Muhialdin B. J. Saari N. Hussin A. S. M. (2020). Review on the biological detoxification of mycotoxins using lactic acid bacteria to enhance the sustainability of foods supply. Molecules 25, 26. 10.3390/MOLECULES25112655 PMC7321335 32517380

[B117] Nian B. Xu Y. J. Liu Y. (2021). Molecular dynamics simulation for mechanism revelation of the safety and nutrition of lipids and derivatives in food: state of the art. Food Res. Int. 145, 110399. 10.1016/J.FOODRES.2021.110399 34112402

[B118] Niederalt C. Kuepfer L. Solodenko J. Eissing T. Siegmund H. U. Block M. (2018). A generic whole body physiologically based pharmacokinetic model for therapeutic proteins in PK-Sim. J. Pharmacokinet. Pharmacodyn. 45, 235–257. 10.1007/S10928-017-9559-4/METRICS 29234936 PMC5845054

[B119] Nunes V. M. Moosavi M. Mousavi Khaneghah A. Oliveira C. A. (2021). Innovative modifications in food processing to reduce the levels of mycotoxins. Curr. Opin. Food Sci. 38, 155–161. 10.1016/J.COFS.2020.11.010

[B120] OECD (n.d.). OECD QSAR Toolbox|OECD. Available online at: https://www.oecd.org/en/data/tools/oecd-qsar-toolbox.html (Accessed July 21, 2025).

[B198] OECD (2004). Synthetic amorphous silica and silicates. Available online at: https://hpvchemicals.oecd.org/ui/handler.axd?id=1db41a5f-cce0-4e6c-bd75-806a9e88a20b . (Accessed July 15, 2025)

[B121] Okus F. Yuzbasioglu D. Unal F. (2024). Molecular docking study of frequently used food additives for selected targets depending on the chromosomal abnormalities they cause. Toxicology 502, 153716. 10.1016/j.tox.2023.153716 38159899

[B122] Onyeaka H. Ghosh S. Obileke K. C. Miri T. Odeyemi O. A. Nwaiwu O. (2024). Preventing chemical contaminants in food: challenges and prospects for safe and sustainable food production. Food Control. 155, 110040. 10.1016/J.FOODCONT.2023.110040

[B123] Otasek D. Morris J. H. Bouças J. Pico A. R. Demchak B. (2019). Cytoscape automation: empowering workflow-based network analysis. Genome Biol. 20, 1–15. 10.1186/S13059-019-1758-4/FIGURES/6 31477170 PMC6717989

[B124] Özel F. Rüegg J. (2023). Exposure to endocrine-disrupting chemicals and implications for neurodevelopment. Dev. Med. Child. Neurol. 65, 1005–1011. 10.1111/DMCN.15551;SUBPAGE:STRING:FULL 36808586

[B125] Páll S. Zhmurov A. Bauer P. Abraham M. Lundborg M. Gray A. (2020). Heterogeneous parallelization and acceleration of molecular dynamics simulations in GROMACS. J. Chem. Phys. 153, 134110. 10.1063/5.0018516/199476 33032406

[B126] Paramasivam A. Murugan R. Jeraud M. Dakkumadugula A. Periyasamy R. Arjunan S. (2024). Additives in processed foods as a potential source of endocrine-disrupting chemicals: a review. J. Xenobiotics 14, 1697–1710. 10.3390/JOX14040090 39584955 PMC11587131

[B127] Pathak R. K. Kim J. M. (2024). Structural insight into the mechanisms and interacting features of endocrine disruptor bisphenol A and its analogs with human estrogen-related receptor gamma. Environ. Pollut. 345, 123549. 10.1016/J.ENVPOL.2024.123549 38350536

[B128] Pathak R. K. Singh D. B. Sagar M. Baunthiyal M. Kumar A. (2020). Computational approaches in drug discovery and design. Computer-Aided Drug Des., 1–21. 10.1007/978-981-15-6815-2_1

[B129] Pathak R. K. Jung D. W. Shin S. H. Ryu B. Y. Lee H. S. Kim J. M. (2024). Deciphering the mechanisms and interactions of the endocrine disruptor bisphenol A and its analogs with the androgen receptor. J. Hazard Mater 469, 133935. 10.1016/J.JHAZMAT.2024.133935 38442602

[B130] Payros D. Dobrindt U. Martin P. Secher T. Bracarense A. P. F. L. Boury M. (2017). The food contaminant deoxynivalenol exacerbates the genotoxicity of gut microbiota. mBio 8, e00007-17. 10.1128/MBIO.00007-17 28292979 PMC5350463

[B131] Peivasteh-roudsari L. Barzegar-bafrouei R. Sharifi K. A. Azimisalim S. Karami M. Abedinzadeh S. (2023). Origin, dietary exposure, and toxicity of endocrine-disrupting food chemical contaminants: a comprehensive review. Heliyon 9, e18140. 10.1016/J.HELIYON.2023.E18140 37539203 PMC10395372

[B197] Perez-Borm P. J. A. Robbins D. Haubold S. Kuhlbusch T. Donaldson K. Schins R. (2006). The potential risks of nanomaterials: a review carried out for ECETOC. Part. Fibre. Toxicol. 3 (11). 16907977 10.1186/1743-8977-3-11 PMC1584248

[B132] Periasamy S. Gopi N. M. (2023). Natural mitigation strategies to control fluoride contamination in agricultural soils. Curr. Opin. Environ. Sci. Health 33, 100467. 10.1016/J.COESH.2023.100467

[B133] Peters R. J. B. Bouwmeester H. Gottardo S. Amenta V. Arena M. Brandhoff P. (2016). Nanomaterials for products and application in agriculture, feed and food. Trends Food Sci. Technol. 9, 155–164. 10.2903/j.efsa.2011.2140

[B134] Piparo E.Lo Worth A. Manibusan M. Yang C. Schilter B. Mazzatorta P. (2011). Use of computational tools in the field of food safety. Regul. Toxicol. Pharmacol. 60, 354–362. 10.1016/J.YRTPH.2011.05.003 21600952

[B135] Pletz J. Blakeman S. Paini A. Parissis N. Worth A. Andersson A. M. (2020). Physiologically based kinetic (PBK) modelling and human biomonitoring data for mixture risk assessment. Environ. Int. 143, 105978. 10.1016/J.ENVINT.2020.105978 32763630 PMC7684529

[B136] Pradeep P. Paul Friedman K. Judson R. (2020). Structure-based QSAR models to predict repeat dose toxicity points of departure. Comput. Toxicol. 16, 100139. 10.1016/J.COMTOX.2020.100139 34017928 PMC8128699

[B137] (Q)SAR (2023). (Q)SAR assessment framework: guidance for the regulatory assessment of (quantitative) structure activity relationship models, predictions, and results based on multiple predictions series on testing and assessment No. 386. Paris: OECD

[B138] Raies A. B. Bajic V. B. (2016). *In silico* toxicology: computational methods for the prediction of chemical toxicity. Wiley Interdiscip. Rev. Comput. Mol. Sci. 6, 147–172. 10.1002/WCMS.1240 27066112 PMC4785608

[B139] Riedmaier A. E. DeMent K. Huckle J. Bransford P. Stillhart C. Lloyd R. (2020). Correction to: use of physiologically based pharmacokinetic (PBPK) modeling for predicting drug-food interactions: an industry perspective. AAPS J. 23, 6. 10.1208/S12248-020-00535-Z 33244667 PMC7852959

[B140] Salo-Ahen O. M. H. Alanko I. Bhadane R. Alexandre A. M. Honorato R. V. Hossain S. (2020). Molecular dynamics simulations in drug discovery and pharmaceutical development. Processes 9, 71. 10.3390/PR9010071

[B141] Salvagni J. Ternus R. Z. Fuentefria A. M. (2011). Assessment of the genotoxic impact of pesticides on farming communities in the countryside of Santa Catarina State, Brazil. Genet. Mol. Biol. 34, 122–126. 10.1590/S1415-47572010005000104 21637554 PMC3085357

[B142] Schacht C. M. Meade A. E. Bernstein A. S. Prasad B. Schlosser P. M. Tran H. T. (2024). Evaluating the impact of anatomical and physiological variability on human equivalent doses using PBPK models. Toxicol. Sci. 200, 241–264. 10.1093/TOXSCI/KFAE067 38796678

[B143] Seal S. Mahale M. García-Ortegón M. Joshi C. K. Hosseini-Gerami L. Beatson A. (2025). Machine learning for toxicity prediction using chemical structures: pillars for success in the real world. Chem. Res. Toxicol. 38, 759–807. 10.1021/ACS.CHEMRESTOX.5C00033/ASSET/IMAGES/LARGE/TX5C00033_0018.JPEG 40314361 PMC12093382

[B144] Sendra M. Štampar M. Fras K. Novoa B. Figueras A. Žegura B. (2023). Adverse (geno)toxic effects of bisphenol A and its analogues in hepatic 3D cell model. Environ. Int. 171, 107721. 10.1016/J.ENVINT.2022.107721 36580735 PMC9875311

[B145] Shan X. Cai Y. Zhu B. Zhou L. Sun X. Xu X. (2024). Rational strategies for improving the efficiency of design and discovery of nanomedicines. Nat. Commun. 15, 9990. 10.1038/s41467-024-54265-3 39557860 PMC11574076

[B146] Sharma R. P. Schuhmacher M. Kumar V. (2018). The development of a pregnancy PBPK model for bisphenol A and its evaluation with the available biomonitoring data. Sci. Total Environ. 624, 55–68. 10.1016/J.SCITOTENV.2017.12.023 29247905

[B147] Sharma A. Ranjit R. Kumar N. Kumar M. Giri B. S. (2023). Nanoparticles based nanosensors: principles and their applications in active packaging for food quality and safety detection. Biochem. Eng. J. 193, 108861. 10.1016/j.bej.2023.108861

[B148] Sherif M. Makame K. R. Östlundh L. Paulo M. S. Nemmar A. Ali B. R. (2023). Genotoxicity of occupational pesticide exposures among agricultural workers in Arab countries: a systematic review and meta-analysis. Toxics 11, 663. 10.3390/TOXICS11080663/S1 37624167 PMC10458041

[B149] Sieg H. Schaar C. Fouquet N. Böhmert L. Thünemann A. F. Braeuning A. (2024). Particulate iron oxide food colorants (E 172) during artificial digestion and their uptake and impact on intestinal cells. Toxicol. Vitro 96, 105772. 10.1016/J.TIV.2024.105772 38199585

[B150] SimBiology (n.d.). SimBiology - MATLAB. Available online at: https://es.mathworks.com/products/simbiology.html (Accessed July 21, 2025).

[B151] Singh A. Vanga S. K. Orsat V. Raghavan V. (2018). Application of molecular dynamic simulation to study food proteins: a review. Crit. Rev. Food Sci. Nutr. 58, 2779–2789. 10.1080/10408398.2017.1341864 28723250

[B152] Smith A. Dong X. Raghavan V. (2022). An overview of molecular dynamics simulation for food products and processes. Processes 10, 119. 10.3390/pr10010119

[B153] Sohal I. S. O’Fallon K. S. Gaines P. Demokritou P. Bello D. (2018). Ingested engineered nanomaterials: state of science in nanotoxicity testing and future research needs. Part Fibre Toxicol. 15, 29. 10.1186/s12989-018-0265-1 29970114 PMC6029122

[B154] Son A. Park J. Kim W. Yoon Y. Lee S. Ji J. (2024). Recent advances in omics, computational models, and advanced screening methods for drug safety and efficacy. Toxics 12, 822. 10.3390/TOXICS12110822 39591001 PMC11598288

[B155] Średnicka P. Juszczuk-Kubiak E. Wójcicki M. Akimowicz M. Roszko M. (2021). Probiotics as a biological detoxification tool of food chemical contamination: a review. Food Chem. Toxicol. 153, 112306. 10.1016/J.FCT.2021.112306 34058235

[B156] Sreedhar D. Manjula N. Pise A. Pise S. Ligade V. S. (2020). Ban of cosmetic testing on animals: a brief overview. Int. J. Curr. Res. Rev. 12, 113–116. 10.31782/IJCRR.2020.121424

[B157] Stenzel M. H. (2021). The trojan horse goes wild: the effect of drug loading on the behavior of nanoparticles. Angew. Chem. Int. Ed. 60, 2202–2206. 10.1002/ANIE.202010934 33210812

[B158] Stoica I. Sadiq S. K. Coveney P. V. (2008). Rapid and accurate prediction of binding free energies for saquinavir-bound HIV-1 proteases. J. Am. Chem. Soc. 130, 2639–2648. 10.1021/JA0779250/SUPPL_FILE/JA0779250_SI 18225901

[B159] Su G. Yu C. Liang S. Wang W. Wang H. (2024). Multi-omics in food safety and authenticity in terms of food components. Food Chem. 437, 137943. 10.1016/J.FOODCHEM.2023.137943 37948800

[B160] Suvarna V. Nair A. Mallya R. Khan T. Omri A. (2022). Antimicrobial nanomaterials for food packaging. Antibiotics 11, 729. 10.3390/ANTIBIOTICS11060729 35740136 PMC9219644

[B161] Suzuki T. Hidaka T. Kumagai Y. Yamamoto M. (2020). Environmental pollutants and the immune response. Nat. Immunol. 21, 1486–1495. 10.1038/S41590-020-0802-6 33046888

[B162] Tang K. H. D. (2025). Genotoxicity of microplastics on living organisms: effects on chromosomes, DNA and gene expression. Environments 12, 10. 10.3390/ENVIRONMENTS12010010

[B163] Tang Y. Qin G. Qian N. Zeng X. Li R. Lai K. P. (2025). Bisphenol A and its replacement chemicals as endocrine disruptors and obesogens. Environ. Chem. Ecotoxicol. 7, 696–705. 10.1016/J.ENCECO.2025.04.001

[B164] The Royal Society Engineering, T. R. A. of Dowling a Clift R. Grobert N. Hutton D. (2004). Nanoscience and nanotechnologies: opportunities and uncertainties, 46. London The Royal Society The Royal Academy of Engineering Report, 618. Available online at: http://scholar.google.com/scholar?hl=en&btnG=Search&q=intitle:Nanoscience+and+nanotechnologies+:+opportunities+and+uncertainties#0.

[B165] Tistaert C. Heimbach T. Xia B. Parrott N. Samant T. S. Kesisoglou F. (2019). Food effect projections *via* physiologically based pharmacokinetic modeling: predictive case studies. J. Pharm. Sci. 108, 592–602. 10.1016/J.XPHS.2018.05.024 29906472

[B166] Tola G. B. (2025). “Food contaminants: a scoping review of sources,” in Toxicity, pathophysiological insights, and mitigation strategies. Basel, Switzerland: Preprints.org 10.20944/preprints202502.0030.v1

[B167] Trisciuzzi D. Alberga D. Leonetti F. Novellino E. Nicolotti O. Mangiatordi G. F. (2018). Molecular docking for predictive toxicology. Methods Mol. Biol. 1800, 181–197. 10.1007/978-1-4939-7899-1_8 29934893

[B168] Troisi G. M. Barton S. J. Liori O. Nyman M. (2020). Polychlorinated biphenyls (PCBs) and sex hormone concentrations in ringed and grey seals: a possible link to endocrine disruption? Arch. Environ. Contam. Toxicol. 78, 513–524. 10.1007/S00244-020-00716-Z/FIGURES/3 32107597 PMC7136188

[B169] Tuccinardi T. (2021). What is the current value of MM/PBSA and MM/GBSA methods in drug discovery? Expert Opin. Drug Discov. 16, 1233–1237. 10.1080/17460441.2021.1942836;WGROUP:STRING:PUBLICATION 34165011

[B170] Usmani S. M. Bremer‐Hoffmann S. Cheyns K. Cubadda F. Dumit V. I. Escher S. E. (2024). Review of new approach methodologies for application in risk assessment of nanoparticles in the food and feed sector: status and challenges. EFSA Support. Publ. 21 (9). 10.2903/sp.efsa.2024.en-8826

[B171] Valdés-Tresanco M. S. Valdés-Tresanco M. E. Valiente P. A. Moreno E. (2021). Gmx_MMPBSA: a new tool to perform end-state free energy calculations with GROMACS. J. Chem. Theory Comput. 17, 6281–6291. 10.1021/ACS.JCTC.1C00645/SUPPL_FILE/CT1C00645_SI_001 34586825

[B172] Valls-Margarit J. Piñero J. Füzi B. Cerisier N. Taboureau O. Furlong L. I. (2023). Assessing network-based methods in the context of system toxicology. Front. Pharmacol. 14, 1225697. 10.3389/FPHAR.2023.1225697/BIBTEX 37502213 PMC10369070

[B173] Vidal-Limon A. Aguilar-Toalá J. E. Liceaga A. M. (2022). Integration of molecular docking analysis and molecular dynamics simulations for studying food proteins and bioactive peptides. J. Agric. Food Chem. 70, 934–943. 10.1021/acs.jafc.1c06110 34990125

[B174] Vorderstrasse B. A. Cundiff J. A. Lawrence B. P. (2004). Developmental exposure to the potent Aryl Hydrocarbon receptor agonist 2,3,7,8-Tetrachlorodibenzo-p-Dioxin impairs the cell-mediated immune response to infection with influenza A virus, but enhances elements of innate immunity. J. Immunotoxicol. 1, 103–112. 10.1080/15476910490509244 18958643

[B175] Vorderstrasse B. A. Cundiff J. A. Lawrence B. P. (2006). A dose-response study of the effects of prenatal and lactational exposure to TCDD on the immune response to influenza A virus. J. Toxicol. Environ. Health A 69, 445–463. 10.1080/15287390500246985;WGROUP:STRING:PUBLICATION 16574621

[B176] Vos J. G. Moore J. A. (1974). Suppression of cellular immunity in rats and mice by maternal treatment with 2, 3, 7, 8-Tetrachlorodibenzo-p-Dioxin. Int. Arch. Allergy Appl. Immunol. 47, 777–794. 10.1159/000231268 4154311

[B177] Wang C. Greene D. Xiao L. Qi R. Luo R. (2018). Recent developments and applications of the MMPBSA method. Front. Mol. Biosci. 4, 1–18. 10.3389/fmolb.2017.00087 29367919 PMC5768160

[B178] Wang Y. Liu T. Xie J. Cheng M. Sun L. Zhang S. (2022). A review on application of molecular simulation technology in food molecules interaction. Curr. Res. Food Sci. 5, 1873–1881. 10.1016/j.crfs.2022.10.012 36276243 PMC9579209

[B179] Wiklund L. Caccia S. Pípal M. Nymark P. Beronius A. (2023). Development of a data-driven approach to adverse outcome pathway network generation: a case study on the EATS-modalities. Front. Toxicol. 5, 1183824. 10.3389/FTOX.2023.1183824/BIBTEX 37229356 PMC10203404

[B180] Winans B. Humble M. C. Lawrence B. P. (2011). Environmental toxicants and the developing immune system: a missing link in the global battle against infectious disease? Reprod. Toxicol. 31, 327–336. 10.1016/J.REPROTOX.2010.09.004 20851760 PMC3033466

[B181] Woo L. L. Egner P. A. Belanger C. L. Wattanawaraporn R. Trudel L. J. Croy R. G. (2011). Aflatoxin B1-DNA adduct Formation and mutagenicity in livers of neonatal Male and female B6C3F1 mice. Toxicol. Sci. 122, 38–44. 10.1093/TOXSCI/KFR087 21507988 PMC3143467

[B182] Wu P. Y. Chou W. C. Wu X. Kamineni V. N. Kuchimanchi Y. Tell L. A. (2024). Development of machine learning-based quantitative structure–activity relationship models for predicting plasma half-lives of drugs in six common food animal species. Toxicol. Sci. 203, 52–66. 10.1093/TOXSCI/KFAE125 39302735

[B183] Xu M. Zhang B. Wang Q. Yuan Y. Sun L. Huang Z. (2018). Theoretical study on the hydrogen bonding interactions in paracetamol-water complexes. J. Chil. Chem. Soc. (Boletín de la Sociedad Chilena de Química), 63, 3788–3794. 10.4067/s0717-97072018000103788

[B184] Yang Y. Faust J. J. Schoepf J. Hristovski K. Capco D. G. Herckes P. (2016). Survey of food-grade silica dioxide nanomaterial occurrence, characterization, human gut impacts and fate across its lifecycle. Sci. Total Environ. 565, 902–912. 10.1016/J.SCITOTENV.2016.01.165 26874640

[B185] Yang D. Yang H. Shi M. Jia X. Sui H. Liu Z. (2023). Advancing food safety risk assessment in China: development of new approach methodologies (NAMs). Front. Toxicol. 5, 1292373. 10.3389/FTOX.2023.1292373/BIBTEX 38046399 PMC10690935

[B186] Yu S. (2024). Formation, occurrence and mitigation strategies of food contaminants and natural toxicants: challenges and prospects. Foods 13, 617. 10.3390/FOODS13040617 38397594 PMC10888199

[B187] Yuan M. Chen S. Zeng C. Fan Y. Ge W. Chen W. (2023). Estrogenic and non-estrogenic effects of bisphenol A and its action mechanism in the zebrafish model: an overview of the past two decades of work. Environ. Int. 176, 107976. 10.1016/J.ENVINT.2023.107976 37236126

[B188] Zahir A. Ge Z. Khan I. A. (2025). Public health risks associated with food process contaminants – a review. J. Food Prot. 88, 100426. 10.1016/J.JFP.2024.100426 39643160

[B189] Zare F. Ataollahi E. Mardaneh P. Sakhteman A. Keshavarz V. Solhjoo A. (2024). A combination of virtual screening, molecular dynamics simulation, MM/PBSA, ADMET, and DFT calculations to identify a potential DPP4 inhibitor. Sci. Rep. 14 (1), 7749. 10.1038/s41598-024-58485-x 38565703 PMC10987597

[B190] Zhang Y. Li J. Yan Y. (2020). Molecular dynamics study of the migration of Bisphenol A from polycarbonate into food simulants. Chem. Phys. Lett. 741, 137125. 10.1016/J.CPLETT.2020.137125

[B191] Zhang W. Ahari H. Zhang Z. Jafari S. M. (2023). Role of silica (SiO_2_) nano/micro-particles in the functionality of degradable packaging films/coatings and their application in food preservation. Trends Food Sci. Technol. 133, 75–86. 10.1016/J.TIFS.2023.01.009

[B192] Zhang S. Chen J. Gao F. Su W. Li T. Wang Y. (2024a). Foodomics as a tool for evaluating food authenticity and safety from field to table: a review. Foods 14, 15. 10.3390/FOODS14010015 39796305 PMC11719641

[B193] Zhang Y. Zhang L. Wu D. Wu Y. Zhang Y. Zhang L. (2024b). Potential provoking effects of environmental pollutants on food allergy: an issue that is gaining increasing attention. China CDC Wkly. 6 (24), 585–588. 10.46234/CCDCW2024.113 38934022 PMC11196885

[B194] Zhang L. Wu C. Wang Q. (2025a). Toxicity of engineered nanoparticles in food: sources, mechanisms, contributing factors, and assessment techniques. J. Agric. Food Chem. 73, 13142–13158. 10.1021/acs.jafc.5c01550 40418745

[B195] Zhang Z. Tell L. A. Lin Z. (2025b). Comparisons of PK-Sim and R program for physiologically based pharmacokinetic model development for broiler chickens and laying hens: meloxicam as a case study. Toxicol. Sci. 205, 28–41. 10.1093/TOXSCI/KFAF016 39932881

[B196] Zhao L. Zhang Z. Su H. Zhang W. Sun J. Li Y. (2025). Molecular docking–QSAR–Kronecker-regularized least squares-based multiple machine learning for assessment and prediction of PFAS–protein binding interactions. J. Hazard Mater 492, 138069. 10.1016/j.jhazmat.2025.138069 40179788

